# A Comparative Study of Milk Fat Extracted from the Milk of Different Goat Breeds in China: Fatty Acids, Triacylglycerols and Thermal and Spectroscopic Characterization

**DOI:** 10.3390/biom12050730

**Published:** 2022-05-22

**Authors:** Sameh A. Korma, Li Li, Wei Wei, Pengzhan Liu, Xinghe Zhang, Ibrahim A. Bakry, Peipei An, Khaled A. E. Abdrabo, Muhammad Faisal Manzoor, Muhammad Umair, Ilaria Cacciotti, José M. Lorenzo, Carlos Adam Conte-Junior

**Affiliations:** 1 School of Food Science and Engineering, South China University of Technology, Guangzhou 510641, China; sameh.hosny@zu.edu.eg (S.A.K.); lpzhan@scut.edu.cn (P.L.); hyzw1110@163.com (P.A.); abdrabo@aun.edu.eg (K.A.E.A.); 201712800025@mail.scut.edu.cn (M.F.M.); 2 Department of Food Science, Faculty of Agriculture, Zagazig University, Zagazig 44519, Egypt; 3 Sino-Singapore International Joint Research Institute, Guangzhou 510000, China; 4 School of Food Science and Technology, Jiangnan University, Wuxi 214122, China; zhangxinghe_wx@163.com; 5 Department of Food and Dairy Technology, Faculty of Technology and Development, Zagazig University, Zagazig 44519, Egypt; ibrahimbkry@zu.edu.ed; 6 Department of Food Science and Engineering, College of Chemistry and Engineering, Shenzhen University, Shenzhen 518060, China; umair@szu.edu.cn; 7 Department of Engineering, INSTM RU, University of Rome “Niccolò Cusano”, 00166 Roma, Italy; ilaria.cacciotti@unicusano.it; 8 Centro Tecnológico de La Carne de Galicia, Avd. Galicia N° 4, Parque Tecnológico de Galicia, San Cibrao das Viñas, 32900 Ourense, Spain; jmlorenzo@ceteca.net; 9 Facultad de Ciencias de Ourense, Área de Tecnología de los Alimentos, Universidade de Vigo, 32004 Ourense, Spain; 10 Center for Food Analysis (NAL), Technological Development Support Laboratory (LADETEC), Federal University of Rio de Janeiro (UFRJ), Cidade Universitária, Rio de Janeiro 21941-598, Brazil; conte@iq.ufrj.br

**Keywords:** Chinese dairy goat, lipid composition, UPCC-Q-TOF-MS, thermal analysis, infrared spectroscopy, infant formula

## Abstract

Goat milk (GM) is an excellent alternative to cow milk and has recently been used in commercial infant formula preparation due to its superior fat composition. Here, the fatty acid (FA) composition, triacylglycerol (TAG) molecular species, thermal behavior and infrared spectra of extracted milk fat from the milk of the two main breeds of dairy goat bred in China (Guanzhong GM (GZG) and Xinong Saanen GM (XSG)) are investigated. Gas chromatography, Fourier-transform infrared spectroscopy, differential scanning calorimetry and ultra-performance convergence chromatography with quadrupole time-of-flight mass spectrometry are applied. The obtained results evidence significant fat compositional differences based on the breed that produced the considered GM. The major FAs in both GM fats were capric (C10:0), myristic (C14:0), palmitic (C16:0), stearic (C18:0) and oleic (C18:1 n-9c). GZG presented a higher content of medium-chain saturated FAs, while XSG had higher unsaturated FAs with higher ratios of L/Ln and n-6/n-3. A total of 339 and 359 TAGs were detected and quantified in GZG and XSG, and the major TAGs were those of m/z 740.6712 (14.10 ± 0.27%) and m/z 684.6094 (10.94 ± 0.02%), respectively. Milk TAGs of GZG and XSG showed 24–54 and 26–54 total acyl carbon numbers with a 0–4 and 0–5 double bond number at 68 and 72 various retention times, respectively. Thermal analysis showed that all GM fat samples melted below normal body temperature. Infrared spectra revealed higher absorption values of GZG milk fat. This study provides valuable information to the dairy industry sector about GM fat produced in China, assessing the appropriateness of Chinese GM fat to be applied in Chinese infant formula.

## 1. Introduction

In recent years, the global population of goats has noticeably grown, surpassing over one billion heads, about 18% of which are in China (more than 12 million), mainly concentrated in Shaanxi, Shandong, Henan, Hebei, Gansu and Xinjiang provinces [1]. This number has mainly increased due to the increasing demand for goat milk (GM) in the past few years [2]. Historically, China did not have a typical dairy goat breed, and the current Chinese dairy goat breeds are the result of crossing exotic and local dairy goats from Europe at the end of the 19th and early 20th centuries. The resulting goat breeds were gradually cultivated through methodical selection and environmental adaptation [3]. Among them, Guanzhong and Xinong Saanen are the major dairy goats in China. They were adopted as the principal dairy goat breeds in the national goat breeds manual. Both are tolerant of local environmental conditions, guaranteeing a large amount of milk production [1,4,5,6].

GM has attracted great attention because of its benefits to humans, particularly to older people and infants, due to its high digestibility and nutritional value (as a superior supply of fatty acids (FAs), amino acids and minerals), in addition to its therapeutic properties [7]. Moreover, it can be utilized as a substitute for bovine milk for patients and infants and also in infant formula (IF) [8]. GM infant formula is manufactured in numerous countries, including New Zealand, Australia, Russia, Korea, Taiwan and China [9]. Milk fat (MF) is one of the most important milk components and one of the specific characteristics of milk and related dairy products. In addition, MF comprises about 400 different FAs, making it the most complicated natural fat [10]. The FA composition of any fresh milk differs according to factors such as breed, feeding practice, the season in which the milk was obtained, location and lactation period [11,12]. Additionally, the dairy goat breed affects the yield of milk composition: for instance, GM fat (GMF) content ranges from 2.3% to 6.9%, an average of 3.3% [13]. Moreover, the presence of breed-dependent variances in the lipid makeup of GMF has been demonstrated [14]. In order to better use GMF and understand its physicochemical properties, it is necessary to examine its composition and the differences between the milk of various breeds of dairy goats through advanced techniques. One of the distinctive techniques is Fourier-transform infrared (FT-IR) spectroscopy, which is rapid and sensitive, requires minimal sample preparation and a minimal amount of solvents and reagents and can be utilized for semiquantitative and qualitative analyses [15]. Nevertheless, FT-IR spectroscopy usage in the MF field is limited. FT-IR spectroscopy has been used to monitor the adulteration of food, such as butter adulteration with chicken fat [16], lard [17] and mutton fat [18], and, in a recent study in 2020, to determine the differences of functional groups present in camel MF from the milk of different camel breeds [19]. Differential scanning calorimetry (DSC) is also receiving great attention in food analysis since it offers a sensitive, fast and reproducible fingerprint tool for describing edible fats and/or oils [20]. Indeed, studies on different MF sources have indicated that the structure and shape of MF triacylglycerol (TAG) molecules are responsible for the MF melting points, crystallization profile and rheological characteristics [21,22]. Furthermore, it is important to use a powerful detection instrument with sensitivity, higher resolution and accuracy to better understand the chemical composition and physical structure of TAG molecules in GMF. TAGs (98.3%) are considered the most abundant constituent of MF and have a significant impact on the nutritional values and sensory parameters (such as texture and flavor) of milk and related dairy products [23]. Additionally, because of the effect of TAG species on physiological and nutritional properties, the structure of TAGs in dietary fats has attracted interest in the past few years. The matrix-assisted laser desorption ionization time-of-flight mass spectrometry technique is one of the current, widespread, applied techniques in lipidomics. It is simple, sensitive and suitable for the rapid screening of both polar and nonpolar lipids [24]. It has been utilized for lipid description in biological samples [25], vegetable oils [26] and milk fat [27,28]. At the same time, ultra-performance convergence chromatography (UPCC) is a valid alternative method to ultra-performance liquid chromatography (UPLC) for analyzing a wide range of analytics with higher separation, such as some basic compounds, herbal extracts and isomers [29,30]. The UPCC system is considered a friendly, environmental and analytical technique due to an extreme reduction in the use of organic solvents. In addition, it provides a high-resolution MS analysis of TAG species with a short analysis time [31]. At the same time, quadrupole time-of-flight mass spectrometry (Q-TOF-MS) is considered an operative instrument for identifying complex lipids due to its exceptional capability in providing detailed mass data and MS portion ion patterns [32]. UPCC, coupled with Q-TOF-MS, has been utilized as an efficient device to separate complex TAG compositions in human MF [33], corn, sunflower and soybean oils [34], olive oils [35], camel MF [19] and lyophilized cow MF [31].

To our knowledge, no previous studies are available on the employment of this technique for the analysis of TAG compositions in GMF. Therefore, the main aim of this study is to increase knowledge about the characterization of fat fractions extracted from the milk of the two main Chinese dairy goat breeds (Guanzhong and Xinong Saanen) using gas chromatography (GC), UPCC-Q-TOF-MS, DSC and FT-IR. Additionally, UPCC is used to separate TAG molecules in GMF for the first time, allowing the identification of many TAG species within a short analysis time.

## 2. Materials and Methods

### 2.1. Milk Sample Collection

In February 2021, fresh GM samples (45 days since the lactation stage) were collected by complete, manual milking at 7–8 a.m., on the same day, from two different dairy goat breeds cultivated in China: Guanzhong dairy goats (GZG) and Xinong Saanen dairy goats (XSG). Eight individual goats of each breed were milked, and milk samples of each goat breed were obtained from the same farm with similar housing conditions in Yangling, Shaanxi province, China. All the individual samples were mixed to obtain three composite samples for each goat breed. Milk samples were placed into sterile plastic containers, frozen at −20 °C and transported in an icebox to the laboratory for total fat extraction. All the goats were 2 years old, were healthy with no acute mastitis or visible clinical disease and fed regularly with a mixture of maize silage, peanut hay and rich and nourishing concentrated feed (consisting of corn, bran, soybean meal and cottonseed meal). Thus, the differences found between the milk of the two goat breeds could be related to genetic variants and metabolic differences.

### 2.2. Chemicals and Reagents

A standard mixture of 37 FAMEs (fatty acid methyl esters) and lipase from porcine pancreas (type II) was purchased from Sigma-Aldrich Chemical Co Ltd. (Shanghai, China). Thin-layer chromatography plates (TLC; 10 × 20 cm; mean pore diameter 60 Å, average particle size 2–25 µm, thickness 500 µm, with dichlorofluorescein) were obtained from Shanghai Shangbang Decoration Materials Co., Ltd. (Shanghai, China). TAG standards, glyceryl trioleate (purity ≥ 99%), 1,3-myristin-2-olein (purity ≥ 99%), 1,3-olein-2-palmitin (purity ≥ 99%), 1(3)-palmitin-2-olein-3(1)-laurin (purity ≥ 99%) and 1(3),2-dioleoyl-3(1)-palmitoyl-sn-glycerols (purity ≥ 99%) were obtained from Larodan Fine Chemicals AB (Malmö, Sweden). Supercritical CO_2_ (purity ≥ 99.999%) was bought from Wuxi Yuantong Company (Wuxi, China). N-hexane, ethanol, acetonitrile and ammonium formate were chromatographically pure and bought from J&K Scientific (Shanghai, China). Deionized water was utilized to prepare all solutions. All other solvents and reagents, such as methanol, chloroform, diethyl ether, potassium hydroxide, anhydrous sodium sulfate and acetic acid, were of chromatographic and analytical grade.

### 2.3. Total Fat Extraction

The extraction of total fat from GM samples was performed according to the Floch method [36]. Briefly, 20 mL of each GM sample was dissolved in 100 mL of chloroform–methanol (2:1, *v*/*v*), magnetically stirred for 15 min and, finally, centrifuged for 10 min at 5000 rpm. The lower organic phase, including the total lipids, was taken in a separation funnel and then mixed with 1/4 volume of a sodium chloride solution (NaCl, 0.86%, *w*/*w*) to be equilibrated. The lower organic layer was separated, filtered and transferred in a rotary vacuum evaporator at 40 °C. The extraction was carried out in an environment with weak light and at room temperature in order to minimize the tendency of lipids to oxidize. Furthermore, the obtained total lipids were flushed under nitrogen gas and stored at −20 °C for further analysis.

### 2.4. Analysis of Extracted Fat Composition

#### 2.4.1. FA Composition

GM samples’ FA composition was analyzed as FAMEs by using an Agilent 7890A GC coupled with a Trace TR-FAME capillary column (60 m × 250 μm × 0.25 μm; Thermo Fisher, Waltham, MA, USA) and a hydrogen flame ionization detector. FAMEs of GMF were prepared as described by Ali et al. [37]. Briefly, 2 mL n-hexane and 0.5 mL KOH–CH_3_OH solution (2 mol L^−1^) were mixed with 20 mg MF in a sealable tube and then 5 mL saturated NaCl solution was added. The obtained mixture was vortexed for 2 min, and the supernatant was collected and dried over Na_2_SO_4_. Ultimately, 1 mL of the obtained FAME solution was analyzed. Before the analysis, the obtained FAME solution was filtered using a 0.22 µm filter membrane. N_2_ was utilized as a carrier gas (flow rate 1.2 mL min^−1^, split ratio 1:100). The temperature was set at 250 °C for the injector and the detector. The initial column temperature was kept for 3 min at 60 °C and then increased to 175 °C (5 °C min^−1^) and isothermally kept for 15 min and then raised to 220 °C (2 °C min^−1^) and kept for 20 min. The FAMEs were identified by comparing the retention times of sample peaks with those of a mixture of 37 FAME standards (Sigma-Aldrich Chemical Co Ltd., Shanghai, China). Subsequently, FAs were quantified by the peak area normalization method, and the results were expressed as peak area percentages [38].

#### 2.4.2. FA Distribution at *sn*-2 and *sn*-1, 3 Positions

Fat extracted from each GM sample was hydrolyzed to 2-monoacylglycerol (2-MAG) following the method of Abed et al. [39]. For each sample, 20 mg pancreatic lipase, 1000 μL Tris-HCl buffer (pH 8.0, 1 mol L^−1^), 250 μL bile salts (0.05%) and 100 μL CaCl_2_ (2.2%) were mixed with 20 mg MF in a sealable tube. The mixture was maintained in a hot water bath for 3 min at 37 °C while being vortexed (3 times) at 200 rpm. After that, 2 mL diethyl ether and 1000 μL HCl solution (6 mol L^−1^) were added to stop the enzyme reaction. Then, the mixture was submitted to centrifugation at 4500 rpm for 5 min. The supernatant was obtained and then diethyl ether was evaporated under N_2_ until 0.5 mL. The hydrolytic product was then extracted and separated on TLC plates with a solvent system based on diethyl ether–hexane–acetic acid (50:50:1, *v*/*v*/*v*). Finally, the 2-MAG band was placed using a UV light then scraped off, separated with diethyl ether, converted to FAMEs and investigated using GC as described above. The relative FA contents at *sn*-1, 3 positions were determined using the following equation of *sn*-1, 3 = (3 × total FA—*sn*-2 FA)/2.

#### 2.4.3. Separation and Identification of TAG Species by UPCC-Q-TOF-MS

The fat samples of each GM breed were diluted for analysis in n-hexane and filtered using a 0.22 µm filter membrane. TAG species of each MF sample were detected with the UPCC system (Waters, Milford, MA, USA) coupled with an ACQUITY UPCC BEH-2EP analytical column (150 mm × 3.0 mm, 1.7 μm, Waters, USA). The column temperature was adjusted to 50 °C. The mobile phase (flow rate 1 mL min^−1^) comprised of (A) supercritical fluid CO_2_ (purity ≥ 99.999%) and (B) ethanol–acetonitrile (1:1, *v*/*v*). A linear gradient elution was set as follows: 2–0.3% B (0–1 min); 0.3–0.6% B (1–18 min); 0.6–2% B (18–18.1 min); and 2% B (2.9 min). Ammonium formate (10 mM) was utilized as a compensation solution (split ratio 1:8, flow rate 0.2 mL min^−1^). For each analysis, the injection volume was 1 μL with a concentration of 1 mg mL^−1^.

The mass spectrometry of fat samples was carried out with a Waters Xevo C2-S Q-TOF-MS (MS; Waters, Milford, MA, USA) in positive ion electrospray ionization (ESI^+^) mode to detect and quantify the TAG species under the following parameters: capillary voltage 3.5 kV; cone voltage 30 eV; ion source temperature 100 °C; desolvation temperature 400 °C; desolvation gas (nitrogen) flow 700 L h^−1^; and collision gas (argon) flow 50 L h^−1^. The raw data of UPCC-Q-TOF-MS were collected and examined with Waters Mass-Lynx software (v4.1) in MSE mode. The low and high collision energies were set as 6 eV and 30–45 eV range, respectively. The scan time in the 200–1500 m/z range for each function was set at 0.5 s.

### 2.5. Physicochemical Characterization of the Extracted Fat

#### 2.5.1. Melting and Crystallization Profiles

The melting and crystallization characteristics of MF samples were acquired employing DSC (Model 214 Polyma, NETZSCH Instruments, Selb, Germany) equipped with an intercooler. Briefly, 8 mg of the MF sample was placed in an aluminum pan (30 μL) and hermetically sealed. A temperature–time program was set as follows: the MF sample was heated from 25 to 80 °C (50 K/min) and maintained for 10 min at 80 °C, then cooled to −65 °C (10 K/min) and kept for 10 min at −65 °C and, in the end, heated to 80 °C (5 K/min). The thermal data were examined through NETZSCH Proteus^®^ software.

#### 2.5.2. Infrared Spectroscopy

MF FT-IR spectra were acquired through an FT-IR spectrometer (VERTEX 70, Bruker Optics, Ettlingen, Germany) at room temperature in the following conditions: wavenumbers range 400–4000 cm^−1^, scans number 32, spectral resolution 4 cm^−1^. The fat samples were homogeneously spread onto the attenuated total reflectance crystal surface. Opus software (version 7.0, Bruker Optics, Ettlingen, Germany) was employed for instrument control and data acquisition.

### 2.6. Statistical Analysis

The relative numbers of FAs and TAG species in GM samples were expressed as means ± standard deviation of three composite samples (n = 3) for the milk of each GM breed. Significant differences were calculated by independent samples *t*-test using the program Microsoft Excel, Windows 2010 (Microsoft Corporation, Redmond, WA, USA). Results were considered statistically significant at a *p*-value ≤ 0.05. *t*-test assuming equal variances was considered with a *p*-value of Levene’s test ≥ 0.05.

## 3. Results and Discussion

### 3.1. FA Compositions and Distribution

The evaluated FA compositions of the GMF samples are displayed in Table 1. Twenty-two FAs were detected and quantified by the peak area normalization method [38]. Since the acids of pentadecylic (C15:0) and margaric acid (C17:0) were detected in the GC chromatograms, and nonadecanoic acid (C19:0) was also found in the subsequent UPCC-Q-TOF-MS analysis, the internal standard was not employed in the analysis. All the quantifications were carried out only using the percentages of the peak areas in the chromatograms and are presented as a relative content (Table 1). Five major FAs (capric (C10:0), myristic (C14:0), palmitic (C16:0), stearic (C18:0) and oleic (C18:1 n-9c); relative content > 6.5%) were in agreement with Park et al. [40]. GMF samples from each breed contained high contents of saturated FAs (SFAs), of which palmitic acid was the most abundant, as reported in previous studies [7,41,42]. In GZG, the major SFAs were palmitic (31.40%), myristic (11.99%), capric (10.36%) and stearic (6.61%) acids, whereas, in XSG, palmitic acid accounted for 23.84% of the total FAs, followed by stearic (13.49%), myristic (9.75%) and capric (9.48%) acids. Furthermore, oleic acid was the predominant unsaturated FA (UFA) in both GMF samples, with 21.79% and 27.18% values for GZG and XSG, respectively. The relative contents of UFAs and monounsaturated FAs in the XSG samples were higher than those in the GZG samples, taking into account the remarkably greater oleic acid amount in the XSG samples. High levels of UFAs are considered preferable since they have the ability to prevent cardiovascular disease [43], and oleic acid not only delivers energy but also lowers the TAGs’ melting point, thus, ensuring the fluidity needed to transport and metabolize the MF globules [44]. The ratio values of L/Ln and n-6/n-3 were higher in the XSG samples, presenting 5.97 and 7.64 values, respectively. A previous study by Bakry et al. [45] pointed out a positive correlation between the ratio values of L/Ln and n-6/n-3; the high L/Ln ratio value was correlated to an increase of the n-6/n-3 ratio. Koletzko et al. [46] recommended that the L/Ln ratio be between 5:1 and 15:1 in IFs because they contend for the same desaturation and elongation enzymes. Furthermore, the French Food Safety Agency recommended that the n-6/n-3 ratio be generally less than 9:1 [47]. Thus, XSG use as an MF substrate for IFs is preferred to GZG. However, more research is still needed for both the L/Ln ratio and n-6/n-3 ratio in the diet. The disparities between the FA profiles in our study are related to the breed, which strongly influences the FA composition in GM [48]. GMF constituents are the most flexible of all solids that exist [49], and, therefore, differences between the milk of different dairy breeds, as well as between individuals in each breed, are usually expected.

The FA distribution of TAGs in MF shows they have an important role in nutrition, function and availability since the digestion, absorption and metabolism of TAGs in human nutrition are related to the FA distribution. Korma et al. [50] reported that the FAs freed from the *sn*-1, 3 positions had different metabolic fates in contrast to those from the *sn*-2 position. As shown in Table 1, palmitic acid (34.11%) was the most abundant FA in GZG at the *sn*-2 position, followed by myristic (18.76%), oleic (15.87%) and capric (9.77%) acids. In XSG, palmitic, oleic, myristic, stearic and capric acids were the major FAs placed at the *sn*-2 position, accounting for 31.65%, 20.86%, 16.98%, 9.65% and 7.88%, respectively. Our study findings agree with Prosser et al. [51], who established that palmitic acid was the most abundant SFA at the *sn*-2 position. Several studies demonstrated that TAGs containing a high level of palmitic acid at the *sn*-2 position improve the conditions of intestinal absorption of FAs and calcium and have several clinical nutrition benefits, such as improving stool consistency and bone matrix quality [52,53]. At the *sn*-1, 3 positions of both GMF samples, palmitic, oleic, capric, myristic and stearic acids were the most abundant FAs: 30.05%, 24.75%, 10.66%, 8.61% and 6.99% and 19.9%4, 30.34%, 10.28%, 6.14% and 15.41% in GZG and XSG, respectively. XSG contained higher quantities of UFAs (particularly oleic acid) at the *sn*-1, 3 positions, making it an excellent MF substrate for addition to IF. In the human MF, TAGs are mainly enriched with SFAs, such as palmitic acid, at the *sn*-2 position and with UFAs, such as oleic acid, at the *sn*-1, 3 positions [54,55]. Thus, this study suggests that XSG can be used as a MF source to produce commercial IF enhanced with oleic acid at the *sn*-1, 3 positions and palmitic acid at the *sn*-2 position. Overall, the differences between the FA compositions and their types presented in our study and those stated in former studies [41,56] may be because of several factors, i.e., the difference in the extraction method, detection method, dairy goat breed, animal diet, season and location.

### 3.2. TAG Molecular Species Profiling

**Figure 1:**
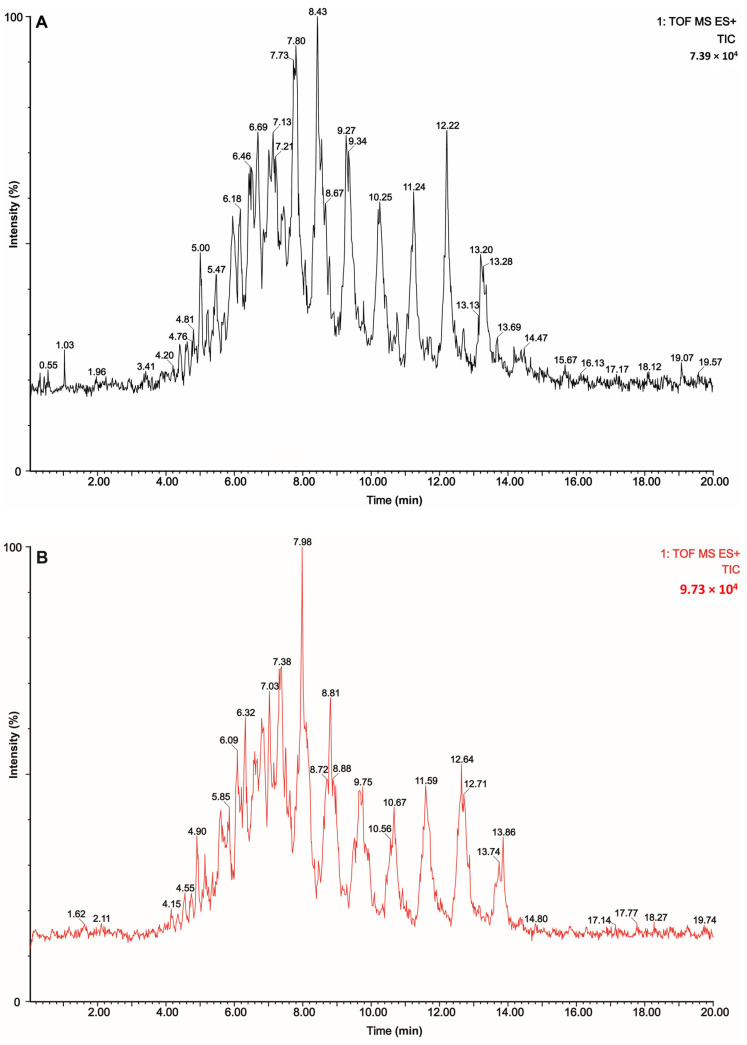
Total ion current chromatograms of TAGs in goat milk fat samples at a collision energy of 35 V analyzed by UPCC-Q-TOF-MS with supercritical carbon dioxide as a mobile phase under the positive ion (ESI^+^) mode within 20 min: (**A**) Guanzhong goat milk, (**B**) Xinong Saanen goat milk.

Knowledge of the GM TAG structures is important for understand their metabolism and physical properties better. Separating and identifying the molecular TAG species in MF are challenging due to their complex structure, especially because these TAGs share the same parent and fragment ions. Therefore, to increase the separation efficiency of TAG molecular species present in GMF extracts, UPCC, followed by the MS detection method, was applied according to our previously developed method [33]. Figure 1 illustrates the total ion current chromatograms of the TAGs in the GMF samples of each breed analyzed using the developed method of UPCC-Q-TOF-MS with supercritical CO_2_ as a mobile phase under ESI^+^ mode.

According to previous studies by Ruiz-Gutiérrez and Barron [57] and Mottram et al. [58], the elution cycle or the order of TAG molecular species in chromatographic methods was mostly related to the partition number (PN). The PN calculation for each TAG was described in previous publications as follows: PN = ACN − 2DB, where ACN is related to the acyl carbon number and DB to the double bonds number in esterified FAs at the glycerol molecule backbone. It was reported that TAGs with higher PN values showed longer retention times compared to those with lower PN values, while the retention times of TAGs that had the same PN values lengthened as the DB increased. However, the identification of milk TAGs on the basis of the PN is complicated because of co-elution occurrence. It has been demonstrated that the presence of long-chain saturated components increases PN values with overlapping PN groups, and the components of unsaturated or very short chains reduce the actual PN [59]. A remarkable PN value decrease was detected when unsaturated FAs occupied the *sn*-2 position, and a rise was noted when very long-chain FAs were placed at an external position. To address such a task and accomplish reliable milk TAG quantification, a fragmentation analysis was performed using Q-TOF-MS of TAGs in ESI^+^ mode [58,59]. The evaluation of TAG and DAG composition was performed by Q-TOF-MSE acquisition data. MSE mode is an efficient method, able to provide two MS scans for data acquisition in each analysis, and, thus, it can offer exact mass data for both quasi-molecular ions and fragment ions in a single injection that is operated via using individually low and high collision energy [37]. The molecular formulas of DAGs and TAGs were accurately established by the mass of the quasi-molecular ions [M + Na]^+^ and [M + NH_4_]^+^, respectively, and the individual FAs on the glyceride molecules were assumed by the [M + H-FA]^+^ fragment ions. In accordance with Lísa et al. [60], fatty acyl chains at the *sn*-1, 3 positions tended to more easily fragment than fatty acyl chains at the *sn*-2 position, and unsaturated fatty acyl chains were more likely to break than saturated acyl chains, making an abundance of [M + H˗FAs]^+^.

Table S1 shows the composition and relative amount (%, calculated by dividing each TAG peak area by the sum of all TAG peak areas within the sample [37]) of the TAG molecular species detected in both GMF samples. There was a difference in the ACN and the DB of the TAGs identified in both GMF samples. GZG milk TAGs exhibited an ACN ranging from 24 to 54, with a DB from 0 to 4 at 68 various retention times, and ACN 42 was the most abundant TAG. On the other hand, XSG milk TAGs possessed an ACN ranging from 26 to 54, containing a DB of 0 to 5 at 72 various retention times, and ACN 38 was the most abundant TAG. Moreover, a total of 339 and 359 various TAG molecular species were successfully detected and quantified in the GZG and XSG samples, respectively, as shown in Table S1. The major TAGs in GZG, with relative content over 4%, were those of m/z 628.5483, m/z 684.6094, m/z 712.6441, m/z 740.6712, m/z 796.7405, m/z 850.7817 and m/z 876.8040, which represent 4.12 ± 0.07, 6.63 ± 0.01, 10.49 ± 0.15, 14.10 ± 0.27, 8.93 ± 0.30, 4.82 ± 0.07 and 4.09 ± 0.31%, respectively. These TAGs account for 53.18 ± 0.42% of the total TAGs detected in GZG. In XSG, the most abundant TAG species were those of m/z 656.5784 (4.12 ± 0.21%), m/z 684.6094 (10.94 ± 0.02%), m/z 712.6441 (9.30 ± 0.40%), m/z 740.6712 (6.15 ± 0.07%), m/z 766.6907 (5.18 ± 0.14%), m/z 792.7107 (4.37 ± 0.19%) and m/z 876.8040 (6.44 ± 0.20%), which represent 46.50 ± 0.03% of the total TAGs determined in the sample. The obtained findings revealed that the dairy goat breed significantly influenced the molecular species structure of TAGs in the GM samples. These results are consistent with Bakry et al. [19], who reported that the dairy camels’ breeds significantly impacted the structure of the TAG molecular species in camel MF.

By using GC, Fontecha et al. [61] characterized TAG species in GM collected from five herds belonging to five different breeders in the Murcia region (Spain) and reported 16 TAG species with a total ACN ranging from 24 to 54, and ACN 42 was the most abundant TAG. In the same way, Smiddy et al. [62] reported 16 TAG species in GM collected from Dutch farms (Holland), with a total ACN between 24 and 54, with ACN 40 TAGs being abundant. By using reverse-phase high-performance liquid chromatography (RP-HPLC), the TAG species of GM collected from the purebred Boer goat (China) were separated and detected by HPLC–atmospheric pressure chemical ionization mass spectrometry (HPLC-APCI-MS). A total of 30 TAGs was recorded with a total ACN ranging between 34 and 52, and the most abundant TAGs were detected at ACN 40 [63]. In addition, Beccaria et al. [64] revealed 165 TAGs with a total ACN ranging between 22 and 54 in GMF collected from the Calabria region (Italy), using UPLC coupled with APCI ion trap time-of-flight-mass spectrometry (UPLC-APCI-IT-TOF-MS). Recently, Zhang et al. [7] characterized TAG species from lyophilized, mature Saanen GMF; a total of 359 molecular types of TAG, containing a total ACN of between 36 and 62, with abundant TAGs from ACN 38, was detected using UPLC connected to Q-TOF high-resolution tandem mass spectrometry (UPLC-Q-TOF-HR-MS/MS). Compared with other reported approaches, our method (UPCC-Q-TOF-MS) could separate and identify the molecular species of TAGs from GMF in a short time of 20 min. Moreover, the UPCC-Q-TOF-MS method allowed the TAG detection and quantification with the same parent ions and overlapping retention times. The disparities between the findings presented in the present study and those stated in former studies may be because of several factors, for example, the difference in the used detection method, dairy goat species, animal diet, season and location, since the GM used in the current research was collected from a local farm in China.

**Figure 2:**
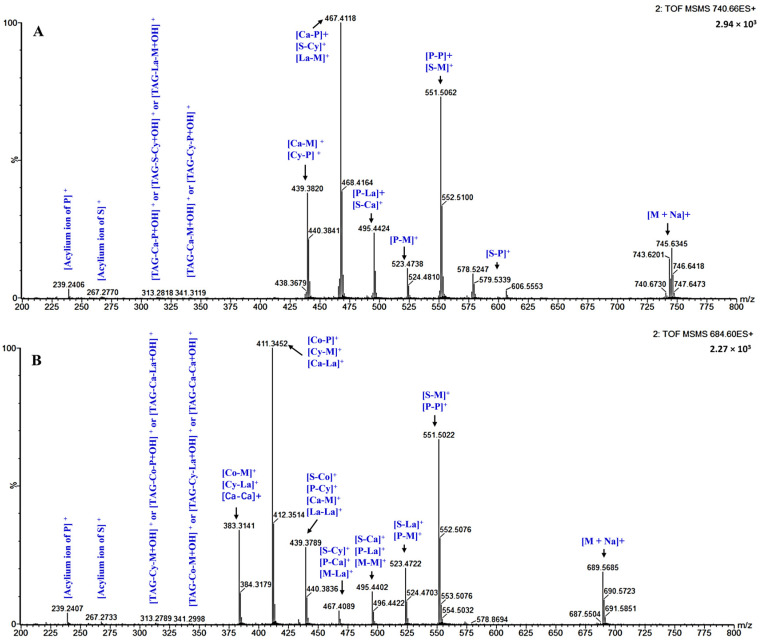
MS^2^ spectra and fragmentations of [M + NH_4_]^+^ for the most abundant TAGs identified in (**A**) Guanzhong goat milk fat, (**B**) Xinong Saanen goat milk fat at m/z 740.6712 and 684.6094, respectively, under the positive ion (ESI^+^) mode. Co, caproic acid (C6:0); Cy, caprylic acid (C8:0); Ca, capric acid (C10:0); La, lauric acid (C12:0); M, myristic acid (C14:0); P, palmitic acid (C16:0); S, stearic acid (C18:0); M + Na, molecular weight + sodium.

Figure 2A,B displays the identification of the MS/MS spectra and fragmentations of the major TAGs detected in the GZG and XSG samples, respectively. Identification of TAGs was achieved using the parental ion in conjunction with the loss of daughter ions available via mass spectrometry [37]. For instance, the multiple TAGs of GZG milk at m/z 740.6712 (Figure 2A) were identified as follows: the daughter ions of S-M-Ca TAG were [Ca-M]^+^:439.3820, [S-Ca]^+^:495.4424 and [S-M]^+^:551.5062, and they were formed from the neutralization of capric, myristic and stearic acids in conjunction with ammonia. The daughter ions of P-P-Ca TAG were [Ca-P]^+^:467.4118 and [P-P]^+^:551.5062 due to the neutralization of capric and palmitic acids in conjunction with ammonia. The daughter ions of S-P-Cy TAG were [Cy-P]^+^:439.3820, [S-Cy]^+^:467.4118 and [S-P]^+^:579.5339, originating from the neutralization of stearic, palmitic and caprylic (C8:0) acids in conjunction with ammonia. The daughter ions of P-M-La TAG were [La-M]^+^:467.4118, [P-La]^+^:495.4424 and [P-M]^+^:523.4738, corresponding to the neutralization of lauric (C12:0), myristic and palmitic acids in conjunction with ammonia. According to Abed et al. [39], the mass spectra of ammoniated TAG [M + NH_4_]^+^ included three main fragment ions as follows: (1) a free FA acylium ion, (2) a protonated monoglyceride ion, obtained after the TAG molecule loses two FAs, and (3) diglyceride (DAG) ions, obtained after losing one FA. For instance, as illustrated in Figure 2A, the MS/MS spectrum of S-M-Ca TAG displayed the corresponding ions: an acylium ion of stearic (S, m/z 267.2770), a protonated monoglyceride ion of [TAG-Ca-M + OH]^+^ (m/z 341.3119) and DAG fragment ions of m/z 439.3820, 495.4424 and 551.5062, which formed because of the neutralization of stearic, myristic and capric acids, respectively. The MS/MS spectrum of P-P-Ca TAG displayed an acylium ion of palmitic (P, m/z 239.2406), a protonated monoglyceride ion of [TAG-Ca-P + OH]^+^ (m/z 313.2818) and DAG fragment ions of m/z 467.4118 and 551.5062, produced because of the neutralization of palmitic and capric acids, respectively. The MS/MS spectrum of S-P-Cy TAG presented an acylium ion of palmitic (P, m/z 239.2406) or an acylium ion of stearic (S, m/z 267.2770), a protonated monoglyceride ion of [TAG-S-Cy + OH]^+^ (m/z 313.2818) or [TAG-Cy-P + OH]^+^ (m/z 341.3119) and DAG fragment ions of m/z 439.3820, 467.4118 and 579.5339, which formed because of the neutralization of stearic, palmitic and caprylic acids, respectively. The MS/MS spectrum of P-M-La TAG exhibited an acylium ion of palmitic (P, m/z 239.2406), a protonated monoglyceride ion of [TAG-La-M + OH]^+^ (m/z 313.2818) and DAG fragment ions of m/z 467.4118, 495.4424 and 523.4738 due to the neutralization of palmitic, myristic and lauric acids, respectively. The identification of the MS/MS spectra and of the fragmentations of the main TAG molecular species in XSG at m/z 684.6094 (Figure 2B) followed the same explanation.

**Figure 3:**
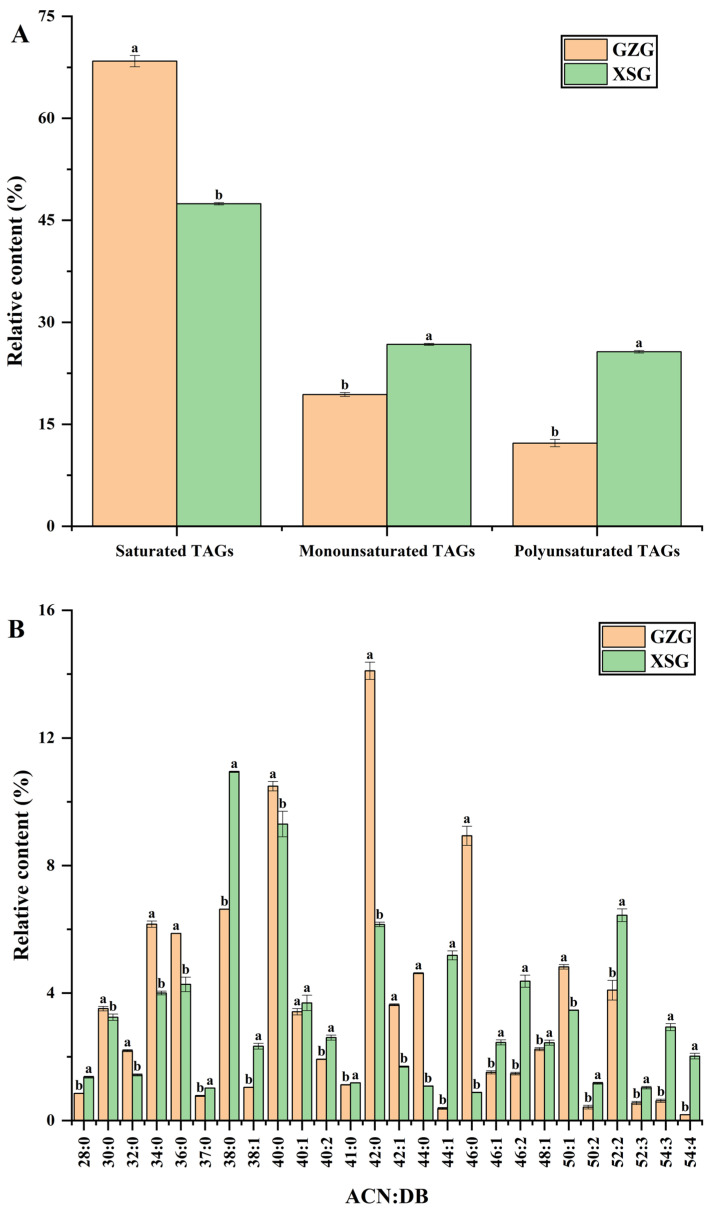
(**A**) Saturation distribution of TAGs; (**B**) Molecular weight distribution of TAGs (>1% of total TAGs) presented as ACN:DB (acyl carbon number:number of double bonds). GZG, Guanzhong goat milk; XSG, Xinong Saanen goat milk. Results represent mean ± standard deviation (indicated by vertical error bars), n = 3. Columns bearing different letters indicate statistical difference (*p* ≤ 0.05) between the two goat dairy breeds by independent samples *t*-test.

As shown in Figure 3A,B, there were significant differences (*p* ≤ 0.05) in the saturation distribution of TAGs and in the molecular weight distribution of the most abundant TAGs with a relative content of over 1% of the total TAGs between the two GM breeds. Saturated TAG content had the highest number of TAGs in both GM samples but was significantly higher in the GZG samples compared to the XSG ones. In general, the saturated TAGs, especially those from ACN 28:0 to ACN 46:0, had large ratios in GM, probably due to the high number of short- and medium-chain FAs in GM compared with other mammalian milk, which promote the therapy of metabolic disorders, anemia and bone demineralization in humans [65]. The TAG molecular species of S-M-Ca, P-P-Ca, S-P-Cy and P-M-La (ACN:DB 42:0) were the predominant saturated TAGs in GZG with a relative abundance of 14.10 ± 0.27%, while, in the XSG samples, S-M-Co, S-La-Cy, S-Ca-Ca, P-P-Co, P-M-Cy, P-La-Ca, M-M-Ca and M-La-La (ACN:DB 38:0) were the major saturated TAGs with a relative content of 10.94 ± 0.02%. In accordance with Cattaneo et al. [66], the consumption of dairy products with a low content of SFAs resulted in a significant reduction in total plasma cholesterol, and most of this reduction was observed in the low-density lipoprotein-cholesterol fraction. In contrast, monounsaturated and polyunsaturated TAGs were rich in XSG samples, with a total of 119 (monounsaturated) and 113 (polyunsaturated) TAGs containing UFAs being detected. The TAGs of m/z 766.6907 (ACN:DB, 44:1) were the richest monounsaturated TAGs, and the TAGs of m/z 876.8040 (ACN:DB, 52:2) were the richest polyunsaturated TAGs. Oleic acid was the most abundant UFA, and it was primarily positioned at the *sn*-1, 3 positions, in accordance with the FAs’ compositions and distribution (Table 1). As the most abundant polyunsaturated TAGs in Chinese and Finnish breast milk [67], the TAGs of O-P-O and L-P-S with ACN:DB 52:2 were the main polyunsaturated TAGs in the GM samples (Table S1 and Figure 3B) and represented more than 4% of total TAGs, in agreement with Zhang et al. [7]. Moreover, Wei et al. [68] reported that TAGs containing oleic acid at the *sn*-1, 3 positions are important for human health and nutrition, as well as for IF formulation. Overall, the TAGs of ACN 42:0 and 38:0 were predominant in GZG and XSG samples, respectively, and these findings are in agreement with some former reports on GMF [7,61].

### 3.3. Melting and Crystallization Thermal Profiles

**Figure 4:**
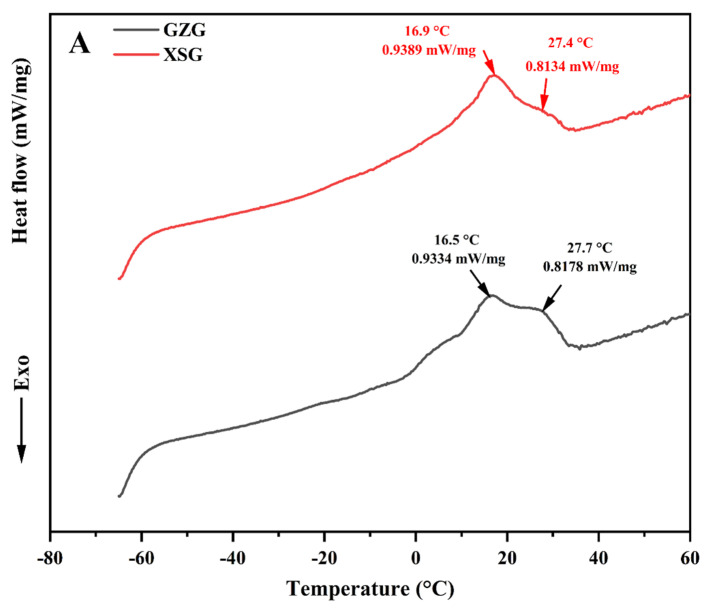

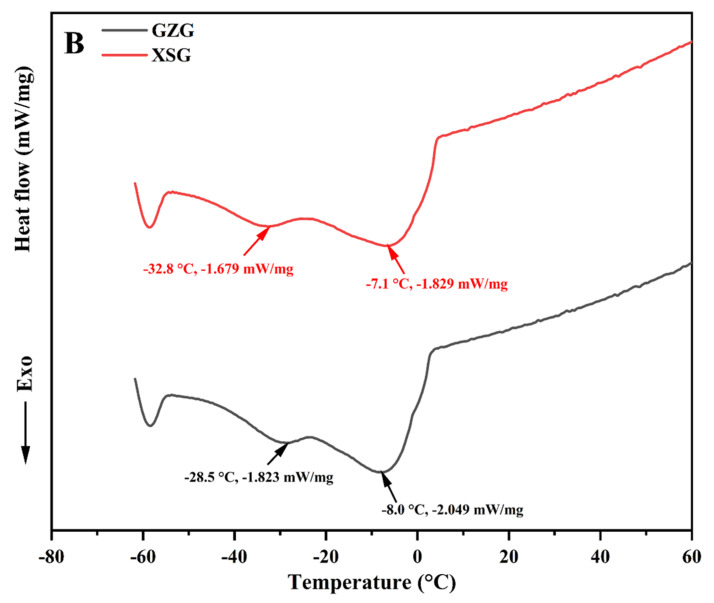
Melting (**A**) and crystallization (**B**) profiles of goat milk fat samples analyzed by differential scanning calorimetry. Guanzhong goat milk (GZG, black curve), Xinong Saanen goat milk (XSG, red curve).

Melting and crystallization thermal behaviors are important indicators for understanding the fats’ physical conditions within the human body, as well as their applications. The differences in the melting and crystallization thermal profiles of MF fractions are attributed to their diverse relative content of FAs and/or TAGs [19]. As shown in Figure 4A, the DSC melting curves presented two endothermic peaks at 16.9 °C and 27.4 °C for XSG and 16.5 °C and 27.7 °C for GZG. These peaks were ascribed to two melting profiles of MF fractions, i.e., middle melting and high melting. Indeed, it has been reported that MF usually exhibits a wide range of melting points varying from −40 to 40 °C because of the large number of TAGs with a large range of chain lengths and saturation degrees. MF frequently displays three overlapping endotherms: low melting with values below 10 °C, middle melting with values between 10 °C and 20 °C and high melting with values above 20 °C [21]. The middle melting temperature of XSG samples was slightly higher (16.9 °C) than that of GZG samples (16.5 °C), probably due to the lower relative content of medium-chain SFAs (Table 1). In contrast, the lower temperature of fat melting (27.4 °C) at the high melting fraction for XSG samples might be due to the higher relative content of monounsaturated FAs (Table 1), particularly oleic acid. Sun et al. [44] stated that a high oleic acid content decreases the TAGs’ melting point, thus, providing the fluidity needed to transport and metabolize the MF globules. In addition, the existence of palmitic acid in the TAG molecular species of the GZG samples and highly saturated TAG molecular species could result in a higher melting temperature at 27.7 °C [52]. Overall, both types of GMF melted below normal human body temperature, which may improve the metabolism and melting at body temperature when included in IF. Moreover, the MF crystallization thermal behavior was also investigated since it is imperative for the processing and texture of dairy products, influencing the spread ability, mouthfeel, appearance and taste [69]. Two crystallization peaks were detected in both breeds; the first point was at −7.1 °C and −8.0 °C, and the second one reached −32.8 °C and −28.5 °C for XSG and GZG, respectively (Figure 4B). The melting and crystallization behaviors found in this study differed from those reported by Sbihi et al. [70] for GMF from Saudi Aradi goats (two melting points at 15.4 °C and 38.7 °C and one crystallization point at 1.9 °C). These disparities may be due to the variation in the GM breed and their FA and TAG compositions.

### 3.4. Infrared Spectroscopy

**Figure 5:**
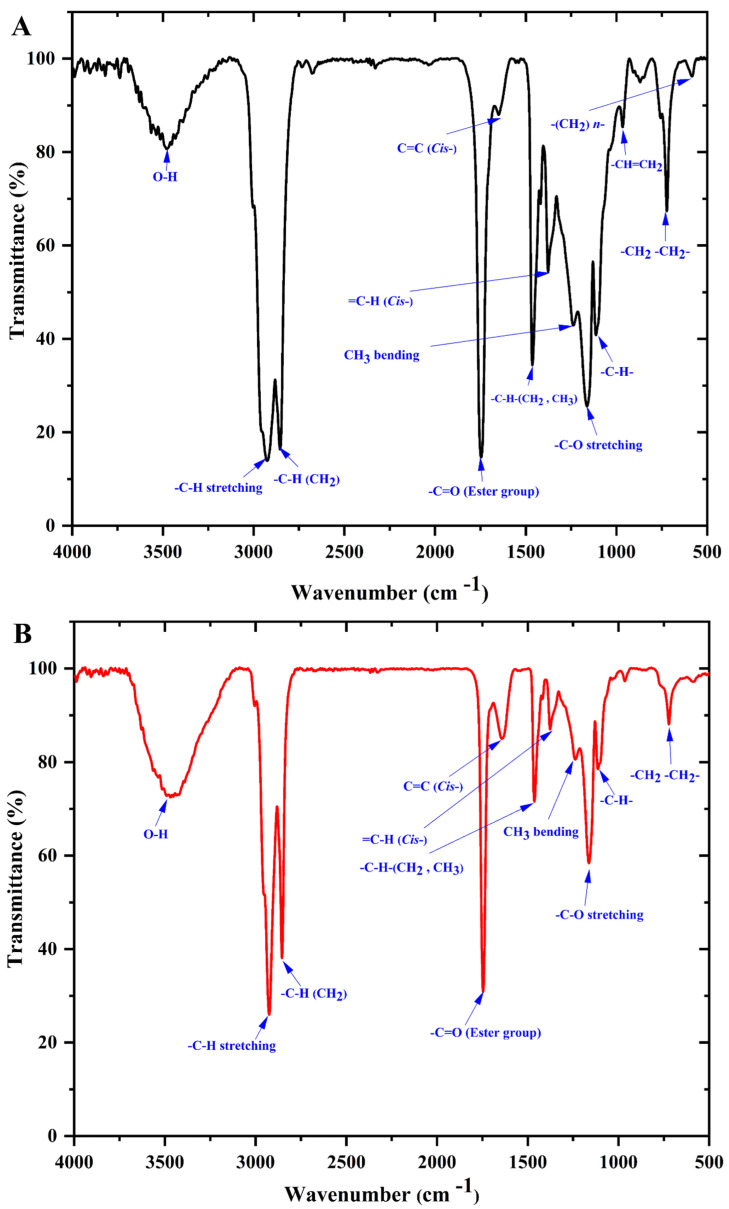
FT-IR spectra of goat milk fat samples fractions in the range of wavenumber 4000–500 cm^−1^; (**A**) Guanzhong goat milk (GZG); (**B**) Xinong Saanen goat milk (XSG).

FT-IR spectra are considered as fingerprint profiles for oils and/or fats since the infrared spectrum absorption in a lipid is caused by existing FAs [71]. As far as we know, there is no research in the literature related to the characterization of and comparison among the spectroscopic patterns of GMF types using FT-IR. Representative FT-IR spectra of the GMF samples extracted from GZG and XSG are exhibited in Figure 5A,B. It can be seen that the FT-IR spectra of the analyzed GMF samples were comparable, except for slight differences in the intensity of the peaks that could be ascribed to the number of FA compositions in each type of GM, with a higher content of the FA types resulting in higher IR absorption [71,72]. The moderate band at 3474 cm^−1^ is related to the overtone of the functional group of -C=O )ester) [73], while the strong absorption peaks at 2924 cm^−1^ and 2852 cm^−1^ relate to the asymmetric and symmetric stretching vibrations of the -C-H (CH_2_) groups, respectively [74]. Moreover, the peaks at 1748 cm^−1^ and 1644 cm^−1^ are ascribed to the stretching of the -C=O (ester) and -C=C- (cis) groups, respectively, as previously reported by Sbihi et al. [70]. The peaks at 1463 cm^−1^ and 1374 cm^−1^ are respectively related to the bending vibrations of the -C-H (CH_2_, CH_3_) and -C-H (CH_3_) symmetric, while the bands at 1237 cm^−1^ and 1165 cm^−1^ are both correlated with the vibration of C-O ester stretching groups and of the CH_2_ bending group, and both are associated with the percentage of saturated acyl groups in the GMF samples [74]. Additionally, the weak band near 1110 cm^−1^ is ascribed to the C-O ester stretching group’s vibration. Finally, the two peaks near 964 cm^−1^ and 724 cm^−1^ are due to -HC=CH-(trans) out of plane bending vibration and the bending vibration of -(CH_2_)n- rocking groups and out-of-plane bending vibration of -HC=CH-(cis), respectively. Sbihi et al. [70] noted that the analyzed FT-IR spectra of Saudi Aradi goat fat were characterized by 15 peaks, whereas, in our study, only 12 peaks were detected. These disparities may be related to the composition of their fat fractions concerning chain length and unsaturation degree. Overall, the integration of the bands derived from the spectra for each milk type confirms the above differences in GMF composition for each breed with higher absorption values for GZG milk fat.

## 4. Conclusions

Our study provides comprehensive information on the FA composition, TAG molecular species, thermal profile and infrared spectra related to GMF extracted from the two main GM breeds bred in China (i.e., Guanzhong and Xinong Saanen), selected on account of its potential nutritional value. Significant differences were observed in the composition and content of the FAs and TAG species between the milk these goat breeds and could be better comprehended by looking for genetic variants. A new method for analyzing TAGs in GMF by UPCC-Q-TOF-MS was applied for the first time. The number of identified and quantified TAGs was higher than in previous studies; thus, the developed UPCC-Q-TOF-MS method is a valuable means for analyzing TAGs in GM and is helpful for a better understanding of the GMF of different Chinese dairy goat breeds. Breeding dairy goats from Xinong Saanen that can produce high amounts of monounsaturated and polyunsaturated TAGs, especially O-P-O (an important structured TAG for infants), may be a good source of GMF for IF preparation. The results of this research provide increased knowledge about GMFs from the two main breeds of dairy goat in China and could be useful for IF manufacture, paving the way for the use of the proposed UPCC-Q-TOF-MS/MS approach for TAG analysis in GMF from other breeds.

## Figures and Tables

**Table 1:** Fatty acid composition (peak area %) and the positional distribution (*sn*-2 and *sn*-1, 3) of fatty acid in goat milk fat samples detected using GC-FID.

Fatty Acids	GZG *			XSG **		
	Total FAs	*sn*-2 FAs	*sn*-1, 3 FAs ^¥^	Total FAs	*sn*-2 FAs	*sn*-1, 3 FAs ^¥^
C4:0	1.05 ± 0.04 ^a^	ND	1.58 ± 0.06 ^a˗^	1.30 ± 0.16 ^a^	ND	1.95 ± 0.24 ^a˗^
C6:0	1.77 ± 0.16 ^a^	ND	2.66 ± 0.24 ^a˗^	1.86 ± 0.08 ^a^	ND	2.79 ± 0.12 ^a˗^
C8:0	2.44 ± 0.08 ^a^	1.23 ± 0.08 ^A^	3.05 ± 0.08 ^a˗^	2.48 ± 0.01 ^a^	1.10 ± 0.08 ^A^	3.17 ± 0.03 ^a˗^
C10:0	10.36 ± 0.16 ^a^	9.77 ± 0.82 ^A^	10.66 ± 0.16 ^a˗^	9.48 ± 0.09 ^b^	7.88 ± 0.01 ^B^	10.28 ± 0.14 ^a˗^
C11:0	0.08 ± 0.03 ^a^	ND	0.13 ± 0.04 ^a˗^	0.09 ± 0.01 ^a^	ND	0.14 ± 0.01 ^a˗^
C12:0	4.67 ± 0.24 ^a^	5.83 ± 0.08 ^A^	4.09 ± 0.33 ^a˗^	3.72 ± 0.02 ^b^	5.71 ± 0.02 ^A^	2.73 ± 0.03 ^b˗^
C13:0	0.40 ± 0.02 ^a^	0.71 ± 0.02 ^A^	0.25 ± 0.01 ^a˗^	0.07 ± 0.01 ^b^	0.09 ± 0.01 ^B^	0.06 ± 0.01 ^b˗^
C14:0	11.99 ± 0.82 ^a^	18.76 ± 0.98 ^A^	8.61 ± 0.73 ^a˗^	9.75 ± 0.09 ^b^	16.98 ± 0.82 ^A^	6.14 ± 0.27 ^b˗^
C14:1 n-5 c	0.38 ± 0.02 ^a^	1.12 ± 0.08	0.01 ± 0.00 ^b˗^	0.19 ± 0.01 ^b^	ND	0.29 ± 0.01 ^a˗^
C15:0	1.09 ± 0.04 ^a^	1.20 ± 0.16 ^A^	1.04 ± 0.02 ^a˗^	0.70 ± 0.16 ^b^	0.97 ± 0.02 ^A^	0.57 ± 0.25 ^a˗^
C15:1 n-5 c	0.48 ± 0.02 ^a^	0.61 ± 0.09	0.42 ± 0.02 ^a˗^	0.24 ± 0.10 ^b^	ND	0.36 ± 0.01 ^b˗^
C16:0	31.40 ± 0.82 ^a^	34.11 ± 0.10 ^A^	30.05 ± 0.78 ^a˗^	23.84 ± 0.82 ^b^	31.65 ± 0.80 ^B^	19.94 ± 0.82 ^b˗^
C16:1 n-7 c	1.33 ± 0.02 ^a^	1.17 ± 0.02 ^A^	1.41 ± 0.03 ^a˗^	1.11 ± 0.01 ^b^	1.14 ± 0.02 ^A^	1.09 ± 0.02 ^b˗^
C17:0	0.72 ± 0.03 ^b^	0.59 ± 0.02 ^B^	0.79 ± 0.04 ^b˗^	0.98 ± 0.02 ^a^	1.09 ± 0.01 ^A^	0.93 ± 0.03 ^a˗^
C17:1 n-7 t	0.41 ± 0.01 ^b^	0.55 ± 0.03 ^B^	0.35 ± 0.00 ^b˗^	0.54 ± 0.02 ^a^	0.81 ± 0.08 ^A^	0.41 ± 0.02 ^a˗^
C18:0	6.61 ± 0.03 ^b^	5.86 ± 0.02 ^B^	6.99 ± 0.04 ^b˗^	13.49 ± 0.82 ^a^	9.65 ± 0.09 ^A^	15.41 ± 1.27 ^a˗^
C18:1 n-9 c	21.79 ± 0.98 ^b^	15.87 ± 0.82 ^B^	24.75 ± 1.06 ^b˗^	27.18 ± 0.81 ^a^	20.86 ± 0.09 ^A^	30.34 ± 1.17 ^a˗^
C18:2 n-6 c	2.54 ± 0.24 ^a^	2.69 ± 0.01 ^A^	2.47 ± 0.36 ^a˗^	1.90 ± 0.08 ^b^	1.83 ± 0.02 ^B^	1.94 ± 0.11 ^a˗^
C18:3 n-6 c	ND	ND	ND	0.30 ± 0.08	ND	0.45 ± 0.12
C18:3 n-3 c	0.64 ± 0.01 ^a^	ND	0.96 ± 0.01 ^a˗^	0.32 ± 0.02 ^b^	ND	0.48 ± 0.02 ^b˗^
C20:0	ND	ND	ND	0.13 ± 0.01	ND	0.20 ± 0.01
C20:4 n-6 c	0.25 ± 0.02 ^a^	ND	0.38 ± 0.02 ^a˗^	0.23 ± 0.02 ^a^	ND	0.35 ± 0.02 ^a˗^
**Sums and ratios of FAs**						
ΣSC-SFAs	2.82 ± 0.20 ^a^	ND	4.24 ± 0.31 ^a˗^	3.16 ± 0.24 ^a^	ND	4.74 ± 0.37 ^a˗^
ΣMC-SFAs	29.94 ± 1.35 ^a^	36.30 ± 1.98 ^A^	26.78 ± 1.03 ^a˗^	25.59 ± 0.22 ^b^	31.76 ± 0.88 ^B^	22.51 ± 0.11 ^b˗^
ΣLC-SFAs	39.82 ± 0.92 ^a^	41.76 ± 1.11 ^A^	38.86 ± 0.83 ^a˗^	39.14 ± 0.16 ^a^	43.36 ± 0.88 ^A^	37.04 ± 0.21 ^b˗^
ΣSFAs	72.58 ± 2.48 ^a^	78.06 ± 3.09 ^A^	69.88 ± 2.17 ^a˗^	67.89 ± 0.62 ^a^	75.12 ± 1.76 ^A^	64.29 ± 0.05 ^b˗^
ΣUFAs	27.82 ± 1.33 ^b^	22.01 ± 1.05 ^B^	30.74 ± 1.47 ^b˗^	32.01 ± 0.96 ^a^	24.64 ± 0.20 ^A^	35.70 ± 1.34 ^a˗^
ΣMUFAs	24.39 ± 1.06 ^b^	19.32 ± 1.04 ^B^	26.93 ± 1.07 ^b˗^	29.26 ± 0.83 ^a^	22.81 ± 0.19 ^A^	32.49 ± 1.16 ^a˗^
ΣPUFAs	3.43 ± 0.27 ^a^	2.69 ± 0.01 ^A^	3.80 ± 0.40 ^a˗^	2.75 ± 0.13 ^b^	1.83 ± 0.02 ^B^	3.21 ± 0.19 ^a˗^
ΣOCS-FAs	1.81 ± 0.07 ^a^	1.79 ± 0.19 ^A^	1.82 ± 0.02 ^a˗^	1.68 ± 0.15 ^a^	2.06 ± 0.01 ^A^	1.49 ± 0.22 ^a˗^
ΣECS-FAs	50.00 ± 1.67 ^a^	58.73 ± 1.90 ^A^	45.64 ± 1.55 ^a˗^	47.08 ± 0.09 ^a^	58.28 ± 1.70 ^A^	41.48 ± 0.72 ^b˗^
Σn-6	2.79 ± 0.26 ^a^	2.69 ± 0.01 ^A^	2.84 ± 0.39 ^a˗^	2.43 ± 0.15 ^a^	1.83 ± 0.02 ^B^	2.73 ± 0.21 ^a˗^
Σn-3	0.64 ± 0.01 ^a^	ND	0.96 ± 0.01 ^a˗^	0.32 ± 0.02 ^b^	ND	0.48 ± 0.02 ^b˗^
n-6/n-3	4.35 ± 0.35 ^b^	ND	2.96 ± 0.37 ^b˗^	7.64 ± 0.85 ^a^	ND	5.73 ± 0.74 ^a˗^
L/Ln	3.96 ± 0.33 ^b^	ND	2.57 ± 0.35 ^b˗^	5.97 ± 0.56 ^a^	ND	4.06 ± 0.45 ^a˗^

* GZG: Guanzhong goat milk; ** XSG: Xinong Saanen goat milk. ^¥^ FA composition at *sn*-1, 3 positions was calculated as (3 × total FA˗*sn*-2)/2. C4:0, butyric acid (Bu); C6:0, caproic acid (Co); C8:0, caprylic acid (Cy); C10:0, capric acid (Ca); C11:0, undecanoic acid (Ud); C12:0, lauric acid (La); C13:0, tridecanoic acid (Tr); C14:0, myristic acid (M); C14:1 n-5c, myristoleic acid (Mo); C15:0, pentadecylic acid (Pe); C15:1 n-5c, pentadecenoic acid (Pen); C16:0, palmitic acid (P); C16:1 n-7c, palmitoleic acid (Po); C17:0, margaric acid (Ma); C17:1 n-7t, heptadecenoic acid (H); C18:0, stearic acid (S); C18:1 n-9c, oleic acid (O); C18:2 n-6c, linoleic acid (L); C18:3 n-6c, gamma-linolenic acid (Gln); C18:3 n-3c, alpha-linolenic acid (Ln); C20:0, arachidic acid (Ar); C20:4 n-6c, arachidonic acid (ARA). Results represent mean ± standard deviation, n = 3. Presented values with different lowercase letters (a, b), uppercase letters (A, B) and lowercase letters (a˗, b˗) in the same row denote significant differences in the FAs of the total, *sn*-2 and *sn*-1, 3 positions, respectively, as obtained by independent samples *t*-test (*p* ≤ 0.05). ND, not detected. ΣSC-SFAs, sum of short-chain saturated fatty acids (C4:0 + C6:0); ΣMC-SFAs, sum of medium-chain saturated fatty acids (C8:0 + C10:0 + C11:0 + C12:0 + C13:0 + C14:0); ΣLC-SFAs, sum of long-chain saturated fatty acids (C15:0 + C16:0 + C17:0 + C18:0 + C20:0); ΣSFAs, sum of saturated fatty acids (C4:0 + C6:0 + C8:0 + C10:0 + C11:0 + C12:0 + C13:0 + C14:0 + C15:0 + C16:0 + C17:0 + C18:0 + C20:0); ΣUFAs, sum of unsaturated fatty acids (C14:1 n-5c + C15:1 n-5c + C16:1 n-7c + C17:1 n-7t + C18:1 n-9c + C18:2 n-6c + C18:3 n-6c + C18:3 n-3c + C20:4 n-6c); ΣMUFAs, sum of monounsaturated fatty acids (one double bond, C14:1 n-5c + C15:1 n-5c + C16:1 n-7c + C17:1 n-7t + C18:1 n-9c); ΣPUFAs, sum of polyunsaturated fatty acids (two or more double bonds, C18:2 n-6c + C18:3 n-6c + C18:3 n-3c + C20:4 n-6c); ΣOCS-FAs, sum of odd chain saturated fatty acids (C15:0 + C17:0); ΣECS-FAs, sum of even chain saturated fatty acids (C14:0 + C16:0 + C18:0); Σn-6, sum of all n-6 fatty acids (C18:2 n-6c + C18:3 n-6c + C20:4 n-6c); Σn-3, sum of all n-3 fatty acids (C18:3 n-3c); n-6/n-3, the ratio between sum of all n-6 and sum of all n-3; L/Ln, the ratio between linoleic acid (C18:2 n-6c) and alpha-linolenic acid (C18:3 n-3c).

## Data Availability

Not applicable.

## Supplementary Materials

The following supporting information can be downloaded at: https://www.mdpi.com/article/10.3390/biom12050730/s1, Table S1: Composition and relative content (%) of TAGs in goat milk fat samples identified according to [M + NH_4_]^+^ by using UPCC-Q-TOF-MS with supercritical carbon dioxide as a mobile phase.

## Abbreviations

GM	Goat milk
GZG	Guanzhong goat milk
XSG	Xinong Saanen goat milk
MF	Milk fat
GMF	Goat milk fat
IF	Infant formula
FAs	Fatty acids
TAGs	Triacylglycerols
PN	Partition number
ACN	Acyl carbon numbers
DB	Double bond number
FT-IR	Fourier-transform infrared spectroscopy
DSC	Differential scanning calorimetry
UPCC	Ultra-performance convergence chromatography
UPLC	Ultra-performance liquid chromatography
Q-TOF-MS	Quadrupole time-of-flight mass spectrometry
RP-HPLC	Reverse-phase high-performance liquid chromatography
APCI-MS	Atmospheric pressure chemical ionization mass spectrometry
HR-MS/MS	High-resolution tandem mass spectrometry
GC	Gas chromatography
FAMEs	Fatty acid methyl esters
TLC	Thin-layer chromatography plates
DAG	Diglyceride
MAG	Monoacylglycerol
SC-SFAs	Short-chain saturated fatty acids
MC-SFAs	Medium-chain saturated fatty acids
LC-SFAs	Long-chain saturated fatty acids
SFAs	Saturated fatty acids
UFAs	Unsaturated fatty acids
MUFAs	Monounsaturated fatty acids
PUFAs	Polyunsaturated fatty acids
OCS-FAs	Odd chain saturated fatty acids
ECS-FAs	Even chain saturated fatty acids
n-6	All n-6 fatty acids
n-3	All n-3 fatty acids
n-6/n-3	The ratio between all n-6 and all n-3
L/Ln	The ratio between C18:2 n-6c and C18:3 n-3c
Bu	Butyric acid (C4:0)
Co	Caproic acid (C6:0)
Cy	Caprylic acid (C8:0)
Ca	Capric acid (C10:0)
De	Decenoic acid (C10:1)
Ud	Undecanoic acid (C11:0)
La	Lauric acid (C12:0)
Lo	Lauroleic acid (C12:1)
Tr	Tridecanoic acid (C13:0)
M	Myristic acid (C14:0)
Mo	Myristoleic acid (C14:1 n-5c)
Pe	Pentadecylic acid (C15:0)
Pen	Pentadecenoic acid (C15:1 n-5c)
P	Palmitic acid (C16:0)
Po	Palmitoleic acid (C16:1 n-7c)
Ma	Margaric acid (C17:0)
H	Heptadecenoic acid (C17:1 n-7t)
S	Stearic acid (C18:0)
O	Oleic acid (C18:1 n-9c)
L	Linoleic acid (C18:2 n-6c)
Gln	Gamma-linolenic acid (C18:3 n-6c)
Ln	Alpha-linolenic acid (C18:3 n-3c)
N	Nonadecanoic acid (C19:0)
No	Nonadecenoic acid (C19:1)
Ar	Arachidic acid (C20:0)
Go	Gadoleic acid (C20:1)
ARA	Arachidonic acid (C20:4 n-6c)
Beh	Behenic acid (C22:0)
Er	Erucic acid (C22:1)
