# Supplementary material for: A Comparative Study of Milk Fat Extracted from the Milk of Different Goat Breeds in China: Fatty Acids, Triacylglycerols and Thermal and Spectroscopic Characterization
Source: Biomolecules. 2022 May 22;12(5):730. doi: 10.3390/biom12050730 (PMC9138446; doi:10.3390/biom12050730)
Supplement: Supplementary file 1 [file biomolecules-12-00730-s001.zip › biomolecules-1692153-supplementary.pdf]

biomolecules

MDPI

Article

A Comparative Study of Milk Fat Extracted from the Milk of Different Goat Breeds in China: Fatty Acids, Triacylglycerols and Thermal and Spectroscopic Characterization

Sameh A. Korma [1,2], Li Li [1,3,*], Wei Wei [4,*], Pengzhan Liu [1], Xinghe Zhang [4], Ibrahim A. Bakry [5], Peipei An [1], Khaled A. E. Abdrabo [1], Muhammad Faisal Manzoor [1], Muhammad Umair [6], Ilaria Cacciotti [7], José M. Lorenzo [8,9] and Carlos Adam Conte-Junior [10]

1 School of Food Science and Engineering, South China University of Technology, Guangzhou 510641, China; sameh.hosny@zu.edu.eg (S.A.K.); lpzhan@scut.edu.cn (P.L.); hyzw1110@163.com (P.A.); abdrabo@aun.edu.eg (K.A.E.A.); 201712800025@mail.scut.edu.cn (M.F.M.)
2 Department of Food Science, Faculty of Agriculture, Zagazig University, 44519 Zagazig, Egypt
3 Sino-Singapore International Joint Research Institute, Guangzhou 510000, China
4 School of Food Science and Technology, Jiangnan University, Wuxi 214122, China; zhangxinghe_wx@163.com
5 Department of Food and Dairy Technology, Faculty of Technology and Development, Zagazig University, 44519 Zagazig, Egypt; ibrahimbkry@zu.edu.ed
6 Department of Food Science and Engineering, College of Chemistry and Engineering, Shenzhen University, Shenzhen 518060, China; umair@szu.edu.cn
7 Department of Engineering, INSTM RU, University of Rome "Niccolò Cusano", 00166 Roma, Italy; ilaria.cacciotti@unicusano.it
8 Centro Tecnológico de La Carne de Galicia, Avd. Galicia N° 4, Parque Tecnológico de Galicia, San Cibrao das Viñas, 32900 Ourense, Spain; jmlorenzo@ceteca.net
9 Facultad de Ciencias de Ourense, Área de Tecnología de los Alimentos, Universidade de Vigo, 32004 Ourense, Spain
10 Center for Food Analysis (NAL), Technological Development Support Laboratory (LADETEC), Federal University of Rio de Janeiro (UFRJ), Cidade Universitária, Rio de Janeiro 21941-598, Brazil; conte@iq.ufrj.br
* Correspondence: lili@scut.edu.cn (L.L.); weiw@jiangnan.edu.cn (W.W.); Tel.: +86-208-711-4262 (L.L.); +86-510-858-767-99 (W.W.)

Table S1. Composition and relative content (%) of TAGs in goat milk fat samples identified according to $[M + NH_4]^+$ by using UPCC-Q-TOF-MS with supercritical carbon dioxide as a mobile phase.

$t_{R\ (min)}$	Mass observed (m/z)	Molecular formula	Identified TAGs	$[DAG]^+$ fragment ions ($[M\text{-}FA + H]^+$)	Relative content (%)		ACN:DB
					GZG *	XSG **	
4.033	488.3759	$C_{27}H_{54}NO_6$+	La-Cy-Bu	[Bu-Cy]$^+$:271.1769; [La-Bu]$^+$:327.2471; [La-Cy]$^+$:383.3165	0.04 ± 0.00	ND	24:0
			Ca-Ca-Bu	[Ca-Bu]$^+$:299.2217; [Ca-Ca]$^+$:383.3165			
			Cy-Cy-Cy	[Cy-Cy]$^+$:327.2471			
4.155	516.4219	$C_{29}H_{58}NO_6$+	P-Co-Bu	[Bu-Co]$^+$:243.1501; [P-Bu]$^+$:383.3159; [P-Co]$^+$:411.3453	0.94 ± 0.02^a	0.21 ± 0.02^b	26:0
			La-Ca-Bu	[Bu-Ca]$^+$:299.2251; [La-Bu]$^+$:327.2584; [La-Ca]$^+$: 411.3453			
			La-Cy-Co	[Co-Cy]$^+$:299.2251; [La-Co]$^+$:355.2927; [La-Cy]$^+$:383.3159			
			Ca-Cy-Cy	[Cy-Cy]$^+$:327.2584; [Ca-Cy]$^+$:355.2927			
			Ca-Ca-Co	[Ca-Co]$^+$:327.2584; [Ca-Ca]$^+$:383.3159			
			M-Cy-Bu	[Bu-Cy]$^+$:271.199; [M-Bu]$^+$:355.2927; [M-Cy]$^+$:411.3453			
			S-Bu-Bu	[S-Bu]$^+$:411.3453; [Bu-Bu]$^+$:215.1276			
			Ud-Bu-Ud	[Ud-Bu]$^+$:313.2854; [Ud-Ud]$^+$:411.3453			
4.345	544.4544	$C_{31}H_{62}NO_6$+	P-Co-Co	[P-Co]$^+$:411.3515; [Co-Co]$^+$:271.1956	0.17 ±0.00^b	0.28 ± 0.01^a	28:0
			La-Cy-Cy	[La-Cy]$^+$:383.3175; [Cy-Cy]$^+$:327.2553			
			Ca-Ca-Cy	[Ca-Cy]$^+$:355.2854; [Ca-Ca]$^+$:383.3175			
			La-Ca-Co	[Co-Ca]$^+$:327.2553; [La-Co]$^+$:355.2854; [La-Ca]$^+$:411.3515			
			Ud-Co-Ud	[Ud-Co]+:341.2854; [Ud-Ud]+: 411.3515			
4.555	544.4552	$C_{31}H_{62}NO_6$+	P-Cy-Bu	[Bu-Cy]$^+$:271.1963; [P-Bu]$^+$:383.3179; [P-Cy]$^+$:439.3792	0.68 ± 0.01^b	1.08 ± 0.02^a	28:0
			M-Ca-Bu	[Bu-Ca]$^+$:299.2235; [M-Bu]$^+$:355.2839; [M-Ca]$^+$:439.3792			
			La-La-Bu	[La-Bu]$^+$:327.2560; [La-La]$^+$:439.3792			
			Ca-Ca-Cy	[Ca-Cy]$^+$:355.2839; [Ca-Ca]$^+$:383.3179			
			La-Cy-Cy	[La-Cy]$^+$:383.3179; [Cy-Cy]$^+$:327.2560			
4.741	572.4848	$C_{33}H_{66}NO_6$+	P-Cy-Co	[Co-Cy]$^+$:299.2246; [P-Co]$^+$:411.3457; [P-Cy]$^+$:439.3794	0.59 ± 0.00^a	0.43 ± 0.02^b	30:0
			M-Ca-Co	[Co-Ca]$^+$:327.2537; [M-Co]$^+$:383.3163; [M-Ca]$^+$:439.3794			
			M-Cy-Cy	[M-Cy]$^+$:411.3457; [Cy-Cy]$^+$:327.2537			
			La-Ca-Cy	[Cy-Ca]$^+$:355.2892; [La-Cy]$^+$:383.3163; [La-Ca]$^+$:411.3457			
			Ca-Ca-Ca	[Ca-Ca]$^+$:383.3163			
			S-Co-Co	[S-Co]$^+$:439.3794; [Co-Co]$^+$:271.1898			
4.902	572.4843	$C_{33}H_{66}NO_6$+	P-Ca-Bu	[Bu-Ca]$^+$:299.2225; [P-Bu]$^+$:383.3154; [P-Ca]$^+$:467.4089	2.92 ± 0.07^a	2.81 ± 0.12^a	30:0
5.056	570.4728	$C_{33}H_{64}NO_6$+	O-Cy-Bu	[Bu-Cy]$^+$:271.1865; [O-Bu]$^+$:409.3291; [O-Cy]$^+$:465.3960	ND	0.05 ± 0.01	30:1
			Po-Ca-Bu	[Bu-Ca]$^+$:299.2219; [Po-Bu]$^+$:381.3013; [Po-Ca]$^+$:465.3960			
5.139	600.5118	$C_{35}H_{70}NO_6$+	P-Ca-Co	[Co-Ca]$^+$:327.25301; [P-Co]$^+$:411.3429; [P-Ca]$^+$:467.4076	0.93 ± 0.01^b	1.31 ± 0.04^a	32:0
			P-Cy-Cy	[Cy-Cy]$^+$:327.25301; [P-Cy]$^+$:439.3754			

			M-La-Co	[Co-La] $^+$:355.2838; [M-Co] $^+$:383.3129; [M-La] $^+$:467.4076			
			M-Ca-Cy	[Cy-Ca]$^+$:355.2838; [M-Cy]$^+$:411.3429; [M-Ca]$^+$:439.3754			
			La-La-Cy	[Cy-La]$^+$:383.3129; [La-La]$^+$:439.3754			
			La-Ca-Ca	[Ca-Ca]$^+$:383.3129; [La-Ca]$^+$:411.3429			
			S-Cy-Co	[Co-Cy]$^+$:299.2258; [S-Co]$^+$:439.3754; [S-Cy]$^+$:467.4076			
5.367	600.5179	$C_{35}H_{70}NO_6$+	P-La-Bu	[Bu-La]$^+$:327.2537; [P-Bu]$^+$:383.3147; [P-La]$^+$:495.4394	1.27 ± 0.02[a]	0.12 ± 0.01[b]	32:0
			M-M-Bu	[Bu-M]$^+$:355.2854; [M-M]$^+$:495.4394			
			S-Ca-Bu	[Bu-Ca]$^+$:299.2253; [S-Bu]$^+$:411.3483; [S-Ca]$^+$:495.4394			
			La-Ca-Ca	[Ca-La]$^+$:411.3483; [Ca-Ca]$^+$:383.3147			
5.495	598.5066	$C_{35}H_{68}NO_6$+	O-Ca-Bu	[Bu-Ca]$^+$:299.2244; [O-Bu]$^+$:409.3343; [O-Ca]$^+$:493.4273	0.18 ± 0.00[a]	0.17 ± 0.01[a]	32:1
			Po-La-Bu	[Bu-La]$^+$:327.2544; [Po-Bu]$^+$:381.3107; [Po-La]$^+$:493.4273			
			Po-Ca-Co	[Co-Ca]$^+$:327.2544; [Po-Co]$^+$:409.3343; [Po-Ca]$^+$:465.4074			
			Mo-La-Co	[Co-La]$^+$:355.2873; [Mo-Co]$^+$:381.3107; [Mo-La]$^+$:465.4074			
			Lo-Ca-Ca	[Lo-Ca]$^+$:409.3343; [Ca-Ca]$^+$:383.3162			
			La-Ca-De	[De-Ca]$^+$:381.3107 ; [La-De]$^+$:409.3343; [La-Ca]$^+$:411.3531			
5.549	614.5391	$C_{36}H_{72}NO_6$+	Ma-Ca-Co	[Co-Ca]$^+$:327.2544; [Ma-Co]$^+$:425.3617; [Ma-Ca]$^+$:481.4270	0.12 ± 0.00[a]	0.12 ± 0.02[a]	33:0
			Ma-Cy-Cy	[Ma-Cy]$^+$:453.3994; [Cy-Cy]$^+$:327.2544			
			Pe-Ca-Cy	[Cy-Ca]$^+$:355.2870; [Pe-Cy]$^+$:425.3617; [Pe-Ca]$^+$:453.3994			
5.604	628.5483	$C_{37}H_{74}NO_6$+	P-La-Co	[Co-La]$^+$:355.28939; [P-Co]$^+$:411.3463; [P-La]$^+$:495.4414	4.12 ± 0.07[a]	2.20 ± 0.08[b]	34:0
			M-Ca-Ca	[M-Ca]$^+$:439.3770; [Ca-Ca]$^+$:383.3161			
			M-M-Co	[M-Co]$^+$:383.3161; [M-M]$^+$:495.4414			
			M-La-Cy	[Cy-La]$^+$:383.3161; [M-Cy]$^+$:411.3463; [M-La]$^+$:467.4114			
			La-La-Ca	[La-Ca]$^+$:411.3463; [La-La]$^+$:439.3770			
			P-Ca-Cy	[Cy-Ca]$^+$:355.28939; [P-Cy]$^+$439.3770; [P-Ca]$^+$:467.4114			
			S-Ca-Co	[Co-Ca]$^+$:327.2541; [S-Co]$^+$:439.3770; [S-Ca]$^+$:495.4414			
5.850	628.5479	$C_{37}H_{74}NO_6$+	P-M-Bu	[Bu-M]$^+$:355.2832; [P-Bu]$^+$:383.315; [P-M]$^+$:523.4709	2.03 ± 0.03[a]	1.81 ± 0.09[b]	34:0
6.004	626.5367	$C_{37}H_{72}NO_6$+	O-La-Bu	[Bu-La]$^+$:327.2559; [O-Bu]$^+$:409.3293; [O-La]$^+$:521.4564	0.41 ± 0.02[a]	0.43 ± 0.04[a]	34:1
			P-Mo-Bu	[Bu-Mo]$^+$:353.2786; [P-Bu]$^+$:383.3224; [P-Mo]$^+$:521.4564			
			P-De-Cy	[Cy-De]$^+$:353.2786; [P-Cy]$^+$:439.3788; [P-De]$^+$:465.3951			
			Po-M-Bu	[Bu-M]$^+$:355.2857; [Po-Bu]$^+$:381.3018; [Po-M]$^+$:521.4564			
			Po-Ca-Cy	[Cy-Ca]$^+$:355.2857; [Po-Cy]$^+$:437.3664; [Po-Ca]$^+$:465.3951			
			Mo-La-Cy	[Cy-La]$^+$:383.3224; [Mo-Cy]$^+$:409.3293; [Mo-La]$^+$:465.3951			
			Mo-Ca-Ca	[Ca-Ca]$^+$:383.3224; [Mo-Ca]$^+$:437.3664			
			La-La-De	[De-La]$^+$:409.3293; [La-La]$^+$:439.3788			
			Lo-M-Cy	[Cy-M]$^+$:411.3304; [Lo-Cy]$^+$:381.3018; [Lo-M]$^+$:465.3951			
			Lo-La-Ca	[Ca-La]$^+$:411.3304; [Lo-Ca]$^+$:409.3293; [Lo-La]$^+$:437.3664			
6.045	642.5677	$C_{38}H_{76}NO_6$+	Ma-La-Co	[Co-La]$^+$:355.2813; [Ma-Co]$^+$:425.3704; [Ma-La]$^+$:509.449	0.40 ± 0.01[a]	0.42 ± 0.02[a]	35:0
			Ma-Ca-Cy	[Cy-Ca]$^+$:355.2813; [Ma-Cy]$^+$:453.3948; [Ma-Ca]$^+$:481.4268			

			P-Tr-Co	[Co-Tr]$^{+}$:369.32967; [P-Co]$^{+}$:411.3529; [P-Tr]$^{+}$:509.449			
			Pe-M-Co	[Co-M]$^{+}$:383.3180; [Pe-Co]$^{+}$:397.3285; [Pe-M]$^{+}$:509.449			
			Pe-La-Cy	[Cy-La]$^{+}$:383.3180; [Pe-Cy]$^{+}$:425.3704; [Pe-La]$^{+}$:481.4268			
			Pe-Ca-Ca	[Ca-Ca]$^{+}$:383.3180; [Pe-Ca]$^{+}$:453.3948			
			Tr-La-Ca	[Ca-La]$^{+}$:411.3529; [Tr-Ca]$^{+}$:425.3704; [Tr-La]$^{+}$:453.3948			
			Ma-M-Bu	[Bu-M]$^{+}$:355.2813; [Ma-Bu]$^{+}$:397.3285; [Ma-M]$^{+}$:537.4912			
			P-Pe-Bu	[Bu-Pe]$^{+}$:369.32967; [P-Bu]$^{+}$:383.3180; [P-Pe]$^{+}$:537.4912			
6.087	656.5759	$C_{39}H_{78}NO_6$+	S-La-Co	[Co-La]$^{+}$:355.2856; [S-Co]$^{+}$:439.3773; [S-La]$^{+}$:523.4716	2.21 ± 0.05^{a}	0.16 ± 0.01^{b}	36:0
			S-Ca-Cy	[Cy-Ca]$^{+}$:355.2856; [S-Cy]$^{+}$:467.4088; [S-Ca]$^{+}$:495.4409			
			P-M-Co	[Co-M]$^{+}$:383.3148; [P-Co]$^{+}$:411.3462; [P-M]$^{+}$:523.4716			
			P-Ca-Ca	[Ca-Ca]$^{+}$:383.3148; [P-Ca]$^{+}$:467.4088			
			La-La-La	[La-La]$^{+}$:439.3773			
			P-La-Cy	[Cy-La]$^{+}$:383.3148; [P-Cy]$^{+}$:439.3773; [P-La]$^{+}$:495.4409			
			M-M-Cy	[Cy-M]$^{+}$:411.3462; [M-M]$^{+}$:495.4409			
			M-La-Ca	[Ca-La]$^{+}$:411.3462; [M-Ca]$^{+}$:439.3773; [M-La]$^{+}$:467.4088			
6.324	656.5784	$C_{39}H_{78}NO_6$+	P-P-Bu	[Bu-P]$^{+}$:383.3160; [P-P]$^{+}$:551.5031	3.66 ± 0.06^{b}	4.12 ± 0.21^{a}	36:0
			S-M-Bu	[Bu-M]$^{+}$:355.2828; [S-Bu]$^{+}$:411.3481; [S-M]$^{+}$:551.5031			
5.561	654.5667	$C_{39}H_{76}NO_6$+	O-M-Bu	[Bu-M]$^{+}$:355.2864; [O-Bu]$^{+}$:409.3323; [O-M]$^{+}$:549.4913	0.31 ± 0.03^{b}	0.73 ± 0.02^{a}	36:1
			P-Ca-De	[De-Ca]$^{+}$:381.3026; [P-De]$^{+}$:465.3986; [P-Ca]$^{+}$:467.3167			
			Po-Ca-Ca	[Ca-Ca]$^{+}$:383.3195; [Po-Ca]$^{+}$:465.3986			
			S-Mo-Bu	[Bu-Mo]$^{+}$:353.2727; [S-Bu]$^{+}$:411.3439; [S-Mo]$^{+}$:549.4913			
6.179	670.6010	$C_{40}H_{80}NO_6$+	Ma-M-Co	[Co-M]$^{+}$:383.3148; [Ma-Co]$^{+}$:425.3634; [Ma-M]$^{+}$:537.4920	0.77 ± 0.02^{b}	1.02 ± 0.00^{a}	37:0
			Ma-La-Cy	[Cy-La]$^{+}$:383.3148; [Ma-Cy]$^{+}$:453.3986; [Ma-La]$^{+}$:509.4580			
			Ma-Ca-Ca	[Ca-Ca]$^{+}$:383.3148; [Ma-Ca]$^{+}$:481.4275			
			P-Pe-Co	[Co-Pe]$^{+}$:397.3317; [P-Co]$^{+}$:411.3488; [P-Pe]$^{+}$:537.4920			
			Pe-M-Cy	[Cy-M]$^{+}$:411.3488; [Pe-Cy]$^{+}$:425.3634; [Pe-M]$^{+}$:509.4580			
			Pe-La-Ca	[Ca-La]$^{+}$:411.3488; [Pe-Ca]$^{+}$:453.3986; [Pe-La]$^{+}$:481.4275			
6.446	668.5842	$C_{40}H_{78}NO_6$+	O-Pe-Bu	[Bu-Pe]$^{+}$:369.3046; [O-Bu]$^{+}$:409.3241; [O-Pe]$^{+}$:563.5032	0.24 ± 0.02^{a}	0.20 ± 0.00^{b}	37:1
			H-P-Bu	[Bu-P]$^{+}$:383.3158; [H-Bu]$^{+}$:395.3169; [H-P]$^{+}$:563.5032			
6.798	684.6094	$C_{41}H_{82}NO_6$+	S-M-Co	[Co-M]$^{+}$:383.3141; [S-Co]$^{+}$:439.3789; [S-M]$^{+}$:551.5022	6.63 ± 0.01^{b}	10.94 ± 0.02^{a}	38:0
			S-La-Cy	[Cy-La]$^{+}$:383.3141; [S-Cy]$^{+}$:467.4089; [S-La]$^{+}$:523.4722			
			S-Ca-Ca	[Ca-Ca]$^{+}$:383.3141; [S-Ca]$^{+}$:495.4402			
			P-P-Co	[Co-P]$^{+}$:411.3452; [P-P]$^{+}$:551.5022			
			P-M-Cy	[Cy-M]$^{+}$:411.3452; [P-Cy]$^{+}$:439.3789; [P-M]$^{+}$:523.4722			
			P-La-Ca	[Ca-La]$^{+}$:411.3452; [P-Ca]$^{+}$:467.4089; [P-La]$^{+}$:495.4402			
			M-M-Ca	[Ca-M]$^{+}$:439.3789; [M-M]$^{+}$:495.4402			
			M-La-La	[La-La]$^{+}$:439.3789; [M-La]$^{+}$:467.4089			
7.026	682.5958	$C_{41}H_{80}NO_6$+	O-P-Bu	[Bu-P]$^{+}$:383.3148; [O-Bu]$^{+}$:409.3311; [O-P]$^{+}$:577.5208	1.04 ± 0.01^{b}	2.33 ± 0.09^{a}	38:1
			Po-Ca-La	[La-Ca]$^{+}$:411.3484; [Po-La]$^{+}$:493.4255; [Po-Ca]$^{+}$:465.3936			
			P-De-La	[La-De]$^{+}$:409.3311; [P-La]$^{+}$:495.4426; [P-De]$^{+}$:465.3936			
			Po-S-Bu	[Bu-S]$^{+}$:411.3484; [Po-Bu]$^{+}$:381.3070; [Po-S]$^{+}$:577.5208			
7.381	712.6441	$C_{43}H_{86}NO_6$+	S-La-Ca	[Ca-La]$^{+}$:411.3467; [S-Ca]$^{+}$:495.4438; [S-La]$^{+}$:523.4278			40:0

			P-M-Ca	[Ca-M]$^+$:439.3803; [P-Ca]$^+$:467.4105; [P-M]$^+$:523.4278	10.49 ± 0.15[a]	9.30 ± 0.40[b]	
			M-M-La	[La-M]$^+$:467.4105; [M-M]$^+$:495.4438			
			Co-S-P	[P-S]$^+$:579.5377; [Co-P]$^+$:411.3467; [Co-S]$^+$:439.3803			
			Cy-P-P	[P-P]$^+$:551.5048; [Cy-P]$^+$:439.3803			
			Bu-S-S	[S-S]$^+$:607.5707; [Bu-S]$^+$:411.3467			
			Ar-Ca-Ca	[Ca-Ca]$^+$:383.3145; [Ar-Ca]$^+$:523.4278			
			Ar-P-Bu	[Bu-P]$^+$:383.3145; [Ar-Bu]$^+$:439.3803; [Ar-P]$^+$:607.5707			
			Ar-La-Cy	[Cy-La]$^+$:383.3145; [Ar-Cy]$^+$:495.4438; [Ar-La]$^+$:551.5048			
7.508	710.6297	$C_{43}H_{84}NO_{6}$+	S-O-Bu	[Bu-O]$^+$:409.3309; [S-Bu]$^+$:411.3476; [S-O]$^+$:605.5447	3.41 ± 0.10[a]	3.69 ± 0.24[a]	40:1
			S-Po-Co	[Co-Po]$^+$:409.3309; [S-Co]$^+$:439.3791; [S-Po]$^+$:577.5255			
			S-Mo-Cy	[Cy-Mo]$^+$:409.3309; [S-Cy]$^+$:467.4131; [S-Mo]$^+$:549.4871			
			S-Lo-Ca	[Ca-Lo]$^+$:409.3309; [S-Ca]$^+$:495.4399; [S-Lo]$^+$:521.4576			
			O-P-Co	[Co-P]$^+$:411.3476; [O-Co]$^+$:437.3657; [O-P]$^+$:577.5255			
			O-M-Cy	[Cy-M]$^+$:411.3476; [O-Cy]$^+$:465.3920; [O-M]$^+$:549.4871			
			O-La-Ca	[Ca-La]$^+$:411.3476; [O-Ca]$^+$:493.4264; [O-La]$^+$:521.4576			
			P-Po-Cy	[Cy-Po]$^+$:437.3657; [P-Cy]$^+$:439.3791; [P-Po]$^+$:549.4871			
			P-Mo-Ca	[Ca-Mo]$^+$:437.3657; [P-Ca]$^+$:467.4131; [P-Mo]$^+$:521.4576			
			P-La-Lo	[Lo-La]$^+$:437.3657; [P-Lo]$^+$:493.4264; [P-La]$^+$:495.4399			
			Po-M-Ca	[Ca-M]$^+$:439.3791; [Po-Ca]$^+$:465.3920; [Po-M]$^+$:521.4576			
			Po-La-La	[La-La]$^+$:439.3791; [Po-La]$^+$:493.4264			
			M-M-Lo	[Lo-M]$^+$:465.3920; [M-M]$^+$:495.4399			
			M-Mo-La	[La-Mo]$^+$:465.3920; [M-La]$^+$:467.4131; [M-Mo]$^+$:493.4264			
			De-M-P	[P-M]$^+$:523.4660; [De-P]$^+$:465.3920; [De-M]$^+$:437.3657			
			De-S-La	[La-S]$^+$:523.4660; [De-La]$^+$:409.3309; [De-S]$^+$:493.4264			
7.855	708.6151	$C_{43}H_{82}NO_{6}$+	O-O-Bu	[Bu-O]$^+$:409.3301; [O-O]$^+$:603.5345	1.92 ± 0.01[b]	2.60 ± 0.08[a]	40:2
			O-Po-Co	[Co-Po]$^+$:409.3301; [O-Co]$^+$:437.3416; [O-Po]$^+$:575.4874			
			O-Mo-Cy	[Cy-Mo]$^+$:409.3301; [O-Cy]$^+$:465.3869; [O-Mo]$^+$:547.4874			
			O-Lo-Ca	[Ca-Lo]$^+$:409.3301; [O-Ca]$^+$:493.4282; [O-Lo]$^+$:519.4453			
			P-Lo-Lo	[Lo-Lo]$^+$:435.3163; [P-Lo]$^+$:493.4282			
			Po-Po-Cy	[Cy-Po]$^+$:437.3416; [Po-Po]$^+$:547.4874			
			Po-Mo-Ca	[Ca-Mo]$^+$:437.3416; [Po-Ca]$^+$:465.3869; [Po-Mo]$^+$:519.4453			
			Po-La-Lo	[Lo-La]$^+$:437.3416; [Po-Lo]$^+$:491.4105; [Po-La]$^+$:493.4282			
			M-Mo-Lo	[Lo-Mo]$^+$:463.3733; [M-Lo]$^+$:465.3869; [M-Mo]$^+$:493.4282			
			Mo-Mo-La	[La-Mo]$^+$:465.3869; [Mo-Mo]$^+$:491.4105			
			Ca-L-La	[La-L]$^+$:519.4453; [Ca-La]$^+$:411.3484; [Ca-L]$^+$:491.4105			
			L-P-Co	[Co-P]$^+$:411.3484; [L-Co]$^+$:435.3163; [L-P]$^+$:575.4874			
			L-M-Cy	[Cy-M]$^+$:411.3484; [L-Cy]$^+$:463.3733; [L-M]$^+$:547.4874			
7.919	706.6003	$C_{43}H_{80}NO_{6}$+	O-L-Bu	[Bu-L]$^+$:407.3161; [O-Bu]$^+$:409.3340; [O-L]$^+$:601.5182	0.28 ± 0.01[b]	0.44 ± 0.07[a]	40:3
7.950	726.6642	$C_{44}H_{88}NO_{6}$+	S-Pe-Cy	[Cy-Pe]$^+$:425.3679; [S-Cy]$^+$:467.4133; [S-Pe]$^+$:565.5246	1.12 ± 0.01[b]	1.18 ± 0.01[a]	41:0
			Ma-P-Cy	[Cy-P]$^+$:439.3772; [Ma-Cy]$^+$:453.3950; [Ma-P]$^+$:565.5246			
			Ma-M-Ca	[Ca-M]$^+$:439.3772; [Ma-Ca]$^+$:481.4189; [Ma-M]$^+$:537.4926			

			P-Pe-Ca	[Ca-Pe]$^+$:453.3950; [P-Ca]$^+$:467.4133; [P-Pe]$^+$:537.4926			
7.982	740.6712	$C_{47}H_{94}NO_6$+	S-M-Ca	[Ca-M]$^+$:439.3820; [S-Ca]$^+$:495.4424; [S-M]$^+$:551.5062	14.10 ± 0.27[a]	6.15 ± 0.07[b]	42:0
			P-P-Ca	[Ca-P]$^+$:467.4118; [P-P]$^+$:551.5062			
			S-P-Cy	[Cy-P]$^+$:439.3820; [S-Cy]$^+$:467.4118; [S-P]$^+$:579.5339			
			P-M-La	[La-M]$^+$:467.4118; [P-La]$^+$:495.4424; [P-M]$^+$:523.4738			
8.100	738.6609	$C_{45}H_{88}NO_6$+	O-P-Cy	[Cy-P]$^+$:439.3777; [O-Cy]$^+$:465.3987; [O-P]$^+$:577.5211	3.63 ± 0.03[a]	1.69 ± 0.02[b]	42:1
			O-M-Ca	[Ca-M]$^+$:439.3777; [O-Ca]$^+$:493.4267; [O-M]$^+$:549.4893			
			O-La-La	[La-La]$^+$:439.3777; [O-La]$^+$:521.4584			
			P-Po-Ca	[Ca-Po]$^+$:465.3987; [P-Ca]$^+$:467.4155; [P-Po]$^+$:549.4893			
			P-M-Lo	[Lo-M]$^+$:465.3987; [P-Lo]$^+$:493.4267; [P-M]$^+$:523.4753			
			P-Mo-La	[La-Mo]$^+$:465.3987; [P-La]$^+$:495.4426; [P-Mo]$^+$:521.4584			
			Po-M-La	[La-M]$^+$:467.4155; [Po-La]$^+$:493.4267; [Po-M]$^+$:521.4584			
			P-P-De	[De-P]$^+$:465.3987; [P-P]$^+$:551.4984			
			M-S-De	[De-S]$^+$:493.4267; [M-De]$^+$:437.3618; [M-S]$^+$:551.4984			
			Ar-Po-Co	[Co-Po]$^+$:409.3284; [Ar-Co]$^+$:467.4155; [Ar-Po]$^+$:605.5229			
			Ar-Mo-Cy	[Cy-Mo]$^+$:409.3284; [Ar-Cy]$^+$:495.4426; [Ar-Mo]$^+$:577.5211			
			Ar-Lo-Ca	[Ca-Lo]$^+$:409.3284; [Ar-Ca]$^+$:523.4753; [Ar-Lo]$^+$:549.4893			
8.456	754.6943	$C_{46}H_{92}NO_6$+	S-Pe-Ca	[Ca-Pe]$^+$:453.3826; [S-Ca]$^+$:495.4454; [S-Pe]$^+$:565.52	0.05 ± 0.00[b]	0.68 ± 0.00[a]	43:0
			Ma-P-Ca	[Ca-P]$^+$:467.4119; [Ma-Ca]$^+$:481.4261; [Ma-P]$^+$:565.5233			
			Ma-M-La	[La-M]$^+$:467.4119; [Ma-La]$^+$:509.4608; [Ma-M]$^+$:537.4890			
			P-Pe-La	[La-Pe]$^+$:481.4261; [P-La]$^+$:495.4454; [P-Pe]$^+$:537.4890			
			S-Ma-Cy	[Cy-Ma]$^+$:453.3826; [S-Cy]$^+$:467.4119; [S-Ma]$^+$:593.5556			
8.456	752.6806	$C_{46}H_{90}NO_6$+	O-Pe-Ca	[Ca-Pe]$^+$:453.3947; [O-Ca]$^+$:493.4262; [O-Pe]$^+$:563.5102	ND	0.73 ± 0.02	43:1
			H-P-Ca	[Ca-P]$^+$:467.4101; [H-Ca]$^+$:479.4143; [H-P]$^+$:563.5102			
			Po-Pe-La	[La-Pe]$^+$:481.4284; [Po-La]$^+$:493.4262; [Po-Pe]$^+$:535.4798			
			Pen-P-La	[La-P]$^+$:495.4410; [Pen-La]$^+$:479.4143; [Pen-P]$^+$:535.4798			
			Mo-Pe-M	[M-Pe]$^+$:509.4605; [Mo-M]$^+$:493.4262; [Mo-Pe]$^+$:507.4648			
			Pe-P-Lo	[Lo-P]$^+$:493.4262; [Pe-Lo]$^+$:479.4143; [Pe-P]$^+$:537.4852			
			S-Pe-De	[De-Pe]$^+$:451.3820; [S-De]$^+$:493.4262; [S-Pe]$^+$:565.5132			
			S-De-Pen	[Pen-De]$^+$:451.3820; [S-Pen]$^+$:565.5132; [S-De]$^+$:495.4410			
8.574	736.6495	$C_{45}H_{86}NO_6$+	O-M-De	[De-M]$^+$:437.3683; [O-De]$^+$:491.4168; [O-M]$^+$:549.4951	0.03 ± 0.00[b]	0.59 ± 0.01[a]	42:2
			O-Mo-Ca	[Ca-Mo]$^+$:437.3683; [O-Ca]$^+$:493.4271; [O-Mo]$^+$:547.4724			
			L-M-Ca	[Ca-M]$^+$:439.3786; [L-Ca]$^+$:491.4168; [L-M]$^+$:547.4724			
			P-Po-De	[De-Po]$^+$:463.3842; [P-De]$^+$:465.4016; [P-Po]$^+$:549.4951			
			M-Mo-Mo	[Mo-Mo]$^+$:491.4168; [M-Mo]$^+$:493.4271			
			Go-Mo-Cy	[Cy-Mo]$^+$:409.3611; [Go-Cy]$^+$:493.4271; [Go-Mo]$^+$:575.4996			
			S-Mo-De	[De-Mo]$^+$:435.3509; [S-De]$^+$:493.4271; [S-Mo]$^+$:549.4951			
			O-Po-Cy	[Cy-Po]$^+$:437.3683; [O-Cy]$^+$:465.4016; [O-Po]$^+$:575.4996			
			L-P-Cy	[Cy-P]$^+$:439.3786; [L-Cy]$^+$:463.3842; [L-P]$^+$:575.4996			
			Go-P-Co	[Co-P]$^+$:409.3611; [Go-Co]$^+$:463.3842; [Go-P]$^+$:603.5459			

8.646	768.7093	$C_{47}H_{94}NO_6$+	S-P-Ca	[Ca-P]+:467.4120; [S-Ca]+:495.4405; [S-P]+:579.5398	3.56 ± 0.03[a]	0.11 ± 0.01[b]	44:0
			S-S-Cy	[Cy-S]+:467.4120; [S-S]+:607.5645			
			Ar-P-Cy	[Cy-P]+:439.3648; [Ar-Cy]+:495.4405; [Ar-P]+:607.5645			
8.720	768.7053	$C_{47}H_{94}NO_6$+	S-M-La	[La-M]+:467.4118; [S-La]+:523.4743; [S-M]+:551.5044	1.06 ± 0.01[a]	0.97 ± 0.02[b]	44:0
			P-M-M	[M-M]+:495.4416; [P-M]+:523.4743			
8.811	766.6907	$C_{47}H_{92}NO_6$+	O-P-Ca	[Ca-P]+:467.4088; [O-Ca]+:493.4244; [O-P]+:577.5208	0.38 ± 0.03[b]	5.18 ± 0.14[a]	44:1
			O-M-La	[La-M]+:467.4088; [O-La]+:521.4575; [O-M]+:549.4867			
			P-Po-La	[La-Po]+:493.4244; [P-La]+:495.4409; [P-Po]+:549.4867			
			P-M-Mo	[Mo-M]+:493.4244; [P-Mo]+:521.4575; [P-M]+:523.4719			
			Po-M-M	[M-M]+:495.4409; [Po-M]+:521.4575			
			S-P-De	[De-P]+:465.3907; [S-De]+:493.4244; [S-P]+:579.5267			
			S-Mo-La	[La-Mo]+:465.3907; [S-La]+:523.4719; [S-Mo]+:549.4867			
			O-S-Cy	[Cy-S]+:467.4088; [O-Cy]+:465.3907; [O-S]+:605.5510			
			S-Po-Ca	[Ca-Po]+:465.3907; [S-Ca]+:495.4409; [S-Po]+:577.5208			
9.285	764.6772	$C_{47}H_{90}NO_6$+	O-Po-Ca	[Ca-Po]+:465.3941; [O-Ca]+:493.4220; [O-Po]+:575.5020	0.77 ± 0.00[b]	0.88 ± 0.01[a]	44:2
			L-P-Ca	[Ca-P]+:467.4126; [L-Ca]+:491.4115; [L-P]+:575.5020			
			L-S-Cy	[Cy-S]+:467.4126; [L-Cy]+:463.3744 ; [L-S]+:603.5362			
			L-P-De	[De-P]+:467.4126; [L-De]+:491.4115; [L-P]+:577.5207			
			S-Po-De	[De-Po]+:463.3744; [S-De]+:493.4220; [S-Po]+:577.5207			
9.378	762.6606	$C_{47}H_{88}NO_6$+	O-L-Cy	[Cy-L]+:463.3793; [O-Cy]+:465.397; [O-L]+:601.5299	0.24 ± 0.01[a]	0.21 ± 0.00[b]	44:3
			O-Po-De	[De-Po]+:463.3793; [O-De]+:491.4095; [O-Po]+:575.4979			
			L-P-De	[De-P]+:465.397; [L-De]+:489.3885; [L-P]+:575.4979			
			L-Po-Ca	[Ca-Po]+:465.397; [L-Ca]+:491.4095; [L-Po]+:573.4914			
			Ln-P-Ca	[Ca-P]+:467.4094; [Ln-Ca]+:489.3885; [Ln-P]+:573.4914			
9.425	782.7233	$C_{48}H_{96}NO_6$+	S-Ma-Ca	[Ca-Ma]+:481.4243; [S-Ca]+:495.4471; [S-Ma]+:593.5403	0.37 ± 0.01[b]	0.43 ± 0.02[a]	45:0
			S-Pe-La	[La-Pe]+:481.4243; [S-La]+:523.4765; [S-Pe]+:565.5621			
			Ma-P-La	[La-P]+:495.4471; [Ma-La]+:509.4581; [Ma-P]+:565.5621			
			P-Pe-M	[M-Pe]+:509.4581; [P-M]+:523.4765; [P-Pe]+:537.4824			
9.448	780.7103	$C_{48}H_{94}NO_6$+	O-Ma-Ca	[Ca-Ma]+:481.4223; [O-Ca]+:493.4251; [O-Ma]+:591.5336	0.24 ± 0.00[b]	0.89 ± 0.01[a]	45:1
			O-Pe-La	[La-Pe]+:481.4223; [O-La]+:521.4601; [O-Pe]+:563.4905			
			Ma-Po-La	[La-Po]+:493.4251; [Ma-La]+:509.4567; [Ma-Po]+:563.4905			
			Ma-M-Mo	[Mo-M]+:493.4251; [Ma-Mo]+:535.4616; [Ma-M]+:537.4751			
			H-P-La	[La-P]+:495.4432; [H-La]+:507.4404; [H-P]+:563.4905			
			H-M-M	[M-M]+:495.4432; [H-M]+:535.4616			
9.471	796.7405	$C_{49}H_{96}NO_6$+	S-P-La	[La-P]+:495.4413; [S-La]+:523.4727; [S-P]+:579.5362	8.93 ± 0.30[a]	0.88 ± 0.01[b]	46:0
			P-P-M	[M-P]+:523.4727; [P-P]+:551.5046			
			Ar-P-Ca	[Ca-P]+:467.4126; [Ar-Ca]+:523.4727; [Ar-P]+:607.5650			
			Beh-M-Ca	[Ca-M]+:439.3787; [Beh-Ca]+:551.5046; [Beh-M]+:607.5650			
9.663	794.7227	$C_{49}H_{96}NO_6$+	S-O-Ca	[Ca-O]+:493.4302; [S-Ca]+:495.4447; [S-O]+:605.5582	1.18 ± 0.04[a]	1.28 ± 0.05[a]	46:1
9.754	794.7258	$C_{49}H_{96}NO_6$+	O-P-La	[La-P]+:495.44409; [O-La]+:521.4586; [O-P]+:577.5190	0.32 ± 0.01[b]		46:1

			O-M-M	[M-M]$^+$:495.44409; [O-M]$^+$:549.4893		1.18 ± 0.03[a]	
			P-Po-M	[M-Po]$^+$:521.4586; [P-M]$^+$:523.4734; [P-Po]$^+$:549.4893			
			P-P-Mo	[Mo-P]$^+$:521.4586; [P-P]$^+$:551.5009			
			Go-M-La	[La-M]$^+$:467.4145; [Go-La]$^+$:549.4893; [Go-M]$^+$:577.5190			
9.909	792.7107	$C_{49}H_{94}NO_6$+	O-O-Ca	[Ca-O]$^+$:493.4272; [O-O]$^+$:603.5304	1.47 ± 0.04[b]	4.37 ± 0.19[a]	46:2
			O-Po-La	[La-Po]$^+$:493.4272; [O-La]$^+$:521.4590; [O-Po]$^+$:575.5083			
			P-Po-Mo	[Mo-Po]$^+$:519.4435; [P-Mo]$^+$:521.4590; [P-Po]$^+$:549.4917			
			O-Mo-M	[M-Mo]$^+$:493.4272; [O-M]$^+$:549.4917; [O-Mo]$^+$:547.4738			
			S-Po-Lo	[Lo-Po]$^+$:491.3987; [S-Lo]$^+$:521.4590; [S-Po]$^+$:577.5272			
			S-O-De	[De-O]$^+$:491.3987; [S-De]$^+$:493.4272; [S-O]$^+$:605.5482			
			Po-Po-M	[M-Po]$^+$:521.4590; [Po-Po]$^+$:547.4738			
			S-Mo-Mo	[Mo-Mo]$^+$:491.3987; [S-Mo]$^+$:549.4917			
			L-P-La	[La-P]$^+$:495.4438; [L-La]$^+$:519.4435; [L-P]$^+$:575.5083			
			L-M-M	[M-M]$^+$:495.4438; [L-M]$^+$:547.4738			
10.332	790.6936	$C_{49}H_{92}NO_6$+	O-O-De	[De-O]$^+$:491.4067; [O-O]$^+$:603.5139	0.11 ± 0.00[b]	0.44 ± 0.02[a]	46:3
			O-L-Ca	[Ca-L]$^+$:491.4067; [O-Ca]$^+$:493.4273; [O-L]$^+$:601.5186			
			O-Po-Lo	[Lo-Po]$^+$:491.4067; [O-Lo]$^+$:519.4462; [O-Po]$^+$:575.4924			
			Ln-S-Ca	[Ca-S]$^+$:495.4396; [Ln-Ca]$^+$:489.3986; [Ln-S]$^+$:601.5186			
			L-S-De	[De-S]$^+$:493.4273; [L-De]$^+$:489.3986; [L-S]$^+$:603.5139			
			Po-Po-Mo	[Mo-Po]$^+$:519.4462; [Po-Po]$^+$:547.4758			
10.447	810.7181	$C_{50}H_{100}NO_6$+	S-Pe-M	[M-Pe]$^+$:509.5443; [S-M]$^+$:551.5043; [S-Pe]$^+$:565.5245	0.37 ± 0.03[a]	0.19 ± 0.01[b]	47:0
			Ma-P-M	[M-P]$^+$:523.4802; [Ma-M]$^+$:537.4909; [Ma-P]$^+$:565.5245			
10.504	808.7416	$C_{50}H_{98}NO_6$+	No-M-M	[M-M]$^+$:495.4455; [No-M]$^+$:563.4995	0.35 ± 0.03[b]	0.43 ± 0.00[a]	47:1
			O-Pe-M	[M-Pe]$^+$:509.4578; [O-M]$^+$:549.4913; [O-Pe]$^+$:563.4995			
			Ma-Po-M	[M-Po]$^+$:521.4575; [Ma-M]$^+$:537.4875; [Ma-Po]$^+$:563.4995			
			Ma-Pe-Pen	[Pen-Pe]$^+$:521.4575; [Ma-Pen]$^+$:549.4913; [Ma-Pe]$^+$:551.5023			
			H-Pe-Pe	[Pe-Pe]$^+$:523.4760; [H-Pe]$^+$:549.4913			
			P-Po-Pe	[Pe-Po]$^+$:535.4694; [P-Pe]$^+$:537.4875; [P-Po]$^+$:549.4913			
10.562	824.7719	$C_{51}H_{102}NO_6$+	S-P-M	[M-P]$^+$:523.4703; [S-M]$^+$:551.5020; [S-P]$^+$:579.5310	0.03 ± 0.00[b]	0.09 ± 0.00[a]	48:0
			P-P-P	[P-P]$^+$:551.5020			
			Ar-P-La	[La-P]$^+$:495.4424; [Ar-La]$^+$:551.5020; [Ar-P]$^+$:607.5682			
			Ar-M-M	[M-M]$^+$:495.4424; [Ar-M]$^+$:579.5310			
10.672	822.7561	$C_{51}H_{100}NO_6$+	O-P-M	[M-P]$^+$:523.4706; [O-M]$^+$:549.4868; [O-P]$^+$:577.5182	2.24 ± 0.05[b]	2.44 ± 0.08[a]	48:1
			S-Po-M	[M-Po]$^+$:521.4626; [S-M]$^+$:551.5020; [S-Po]$^+$:577.5182			
			S-Mo-P	[P-Mo]$^+$:521.4626; [S-P]$^+$:579.5309; [S-Mo]$^+$:549.4868			
			P-P-Po	[Po-P]$^+$:549.4868; [P-P]$^+$:551.5020			
			S-S-Lo	[Lo-S]$^+$:521.4626; [S-S]$^+$:607.5341			
10.917	820.7415	$C_{51}H_{98}NO_6$+	O-O-La	[La-O]$^+$:521.4549; [O-O]$^+$:603.5394	0.59 ± 0.03[b]	0.76 ± 0.00[a]	48:2
			O-P-Mo	[Mo-P]$^+$:521.4549; [O-Mo]$^+$:547.4785; [O-P]$^+$:577.5272			
			O-Po-M	[M-Po]$^+$:521.4549; [O-M]$^+$:549.4933; [O-Po]$^+$:575.5081			
			P-Po-Po	[Po-Po]$^+$:547.4785; [P-Po]$^+$:549.4933			

			S-Po-Mo	[Mo-Po]$^+$:519.4585; [S-Mo]$^+$:549.4933; [S-Po]$^+$:577.5272			
			S-O-Lo	[Lo-O]$^+$:519.4585; [S-Lo]$^+$:521.4549; [S-O]$^+$:605.5237			
			L-S-La	[La-S]$^+$:523.4719; [L-La]$^+$:519.4585; [L-S]$^+$:603.5394			
			L-P-M	[M-P]$^+$:523.4719; [L-M]$^+$:547.4785; [L-P]$^+$:575.5081			
11.024	818.7282	$C_{51}H_{96}NO_6$+	O-L-La	[La-L]$^+$:519.4471; [O-La]$^+$:521.4567; [O-L]$^+$:601.5184	0.23 ± 0.01[a]	0.17 ± 0.00[b]	48:3
			O-Po-Mo	[Mo-Po]$^+$:519.4471; [O-Mo]$^+$:547.4750; [O-Po]$^+$:575.5078			
			L-P-Mo	[Mo-P]$^+$:521.4567; [L-Mo]$^+$:545.4654; [L-P]$^+$:575.5078			
			L-Po-M	[M-Po]$^+$:521.4567; [L-M]$^+$:547.4750; [L-Po]$^+$:573.4930			
			Ln-P-M	[M-P]$^+$:523.4727; [Ln-M]$^+$:545.4654; [Ln-P]$^+$:573.4930			
11.077	838.7857	$C_{52}H_{104}NO_6$+	S-Ma-M	[M-Ma]$^+$:537.4852; [S-M]$^+$:551.5049; [S-Ma]$^+$:593.5395	0.09 ± 0.01[b]	0.12 ± 0.00[a]	49:0
			S-P-Pe	[Pe-P]$^+$:537.4852; [S-P]$^+$:579.5416; [S-Pe]$^+$:565.5264			
			Ma-Ma-Pe	[Pe-Ma]$^+$:551.5049; [Ma-Ma]$^+$:579.5416			
11.130	836.7768	$C_{52}H_{102}NO_6$+	S-H-M	[M-H]$^+$:535.7930; [S-M]$^+$:551.5072; [S-H]$^+$:591.5445	0.14 ± 0.00[b]	0.20 ± 0.00[a]	49:1
			O-P-Pe	[Pe-P]$^+$:537.4899; [O-Pe]$^+$:563.5214; [O-P]$^+$:577.5214			
			Ma-H-Pe	[Pe-H]$^+$:549.4925 ; [Ma-Pe]$^+$:551.5072; [Ma-H]$^+$:577.5214			
			H-P-P	[P-P]$^+$:551.5072; [H-P]$^+$:563.5214			
			S-Ma-Mo	[Mo-Ma]$^+$:535.7930; [S-Mo]$^+$:549.4925; [S-Ma]$^+$:593.5431			
			O-S-Tr	[Tr-S]$^+$:537.4899; [O-Tr]$^+$:535.7930; [O-S]$^+$:605.5498			
11.365	834.7649	$C_{52}H_{100}NO_6$+	O-H-M	[M-H]$^+$:535.4683; [O-M]$^+$:549.4814; [O-H]$^+$:589.5240	ND	0.08 ± 0.00	49:2
			O-Po-Pe	[Pe-Po]$^+$:535.4683; [O-Pe]$^+$:563.5098; [O-Po]$^+$:575.4991			
			L-Ma-M	[M-Ma]$^+$:537.4938; [L-M]$^+$:547.4705; [L-Ma]$^+$:589.5240			
			L-P-Pe	[Pe-P]$^+$:537.4938; [L-Pe]$^+$:561.4965; [L-P]$^+$:575.4991			
			H-H-Pe	[Pe-H]$^+$:549.4814; [H-H]$^+$:575.4991			
			H-P-Po	[Po-P]$^+$:549.4814; [H-Po]$^+$:561.4965; [H-P]$^+$:563.5098			
			O-Ma-Mo	[Mo-Ma]$^+$:535.4683; [O-Mo]$^+$:547.4705; [O-Ma]$^+$:591.5600			
11.519	852.8041	$C_{53}H_{106}NO_6$+	S-P-P	[P-P]$^+$:551.5048; [S-P]$^+$:579.5347	0.45 ± 0.03[b]	0.66 ± 0.01[a]	50:0
			Ar-P-M	[M-P]$^+$:523.4764; [Ar-M]$^+$:579.5347; [Ar-P]$^+$:607.5732			
			Beh-M-M	[M-M]$^+$:495.4517; [Beh-M]$^+$:607.5732			
11.593	850.7817	$C_{53}H_{104}NO_6$+	S-O-M	[M-O]$^+$:549.4885; [S-M]$^+$:551.5026; [S-O]$^+$:605.5463	4.82 ± 0.07[a]	3.46 ± 0.01[b]	50:1
			O-P-P	[P-P]$^+$:551.5026; [O-P]$^+$:577.5152			
			Go-P-M	[M-P]$^+$:523.4730; [Go-M]$^+$:577.5152; [Go-P]$^+$:605.5463			
			S-S-Mo	[Mo-S]$^+$:549.4885; [S-S]$^+$:607.5656			
			S-P-Po	[Po-P]$^+$:549.4885; [S-Po]$^+$:577.5152; [S-P]$^+$:579.5328			
11.830	848.7693	$C_{53}H_{102}NO_6$+	O-P-Po	[Po-P]$^+$:549.4889; [O-Po]$^+$:575.5103; [O-P]$^+$:577.5217	0.42 ± 0.06[b]	1.17 ± 0.03[a]	50:2
			O-O-M	[M-O]$^+$:549.4889; [O-O]$^+$:603.5364			
			S-O-Mo	[Mo-O]$^+$:547.4779; [S-Mo]$^+$:549.4889; [S-O]$^+$:605.5458			
11.949	846.7548	$C_{53}H_{100}NO_6$+	O-O-Mo	[Mo-O]$^+$:547.4079; [O-O]$^+$:603.5284	0.08 ± 0.01[b]	0.28 ± 0.01[a]	50:3
			O-L-M	[M-L]$^+$:547.4079; [O-M]$^+$:549.4863; [O-L]$^+$:601.5201			
			L-P-Po	[Po-P]$^+$:549.4863; [L-Po]$^+$:573.4880; [L-P]$^+$:575.5060			
			Ln-P-P	[P-P]$^+$:551.5009; [Ln-P]$^+$:573.4880			
12.068	864.8017	$C_{54}H_{106}NO_6$+	No-Ma-Pe	[Pe-Ma]$^+$:551.5150; [No-Pe]$^+$:577.5186; [No-Ma]$^+$:605.5285	ND	0.26 ± 0.01	51:1

			No-P-P	[P-P]$^+$:551.5150; [No-P]$^+$:591.5280			
			S-O-Pe	[Pe-O]$^+$:563.5051; [S-Pe]$^+$:565.5168; [S-O]$^+$:605.5285			
			O-Ma-P	[P-Ma]$^+$:565.5168; [O-P]$^+$:577.5186; [O-Ma]$^+$:591.5280			
12.251	862.7877	$C_{54}H_{104}NO_6^+$	O-O-Pe	[Pe-O]$^+$:563.5052; [O-O]$^+$:603.5369	0.05 ± 0.01[b]	0.10 ± 0.00[a]	51:2
			O-Ma-Po	[Po-Ma]$^+$:563.5052; [O-Po]$^+$:575.4844; [O-Ma]$^+$:591.5364			
			O-H-P	[P-H]$^+$:563.5052; [O-P]$^+$:577.5157; [O-H]$^+$:589.5279			
			L-Ma-P	[P-Ma]$^+$:565.5178; [L-P]$^+$:575.4844; [L-Ma]$^+$:589.5279			
			No-H-Pe	[Pe-H]$^+$:549.4899; [No-Pe]$^+$:577.5157; [No-H]$^+$:603.5369			
			No-Ma-Pen	[Pen-Ma]$^+$:549.4899; [No-Pen]$^+$:575.4844; [No-Ma]$^+$:605.5588			
			No-S-Mo	[Mo-S]$^+$:549.4899; [No-Mo]$^+$:561.4742; [No-S]$^+$:619.7220			
			No-O-M	[M-O]$^+$:549.4899; [No-M]$^+$:563.5052; [No-O]$^+$:617.5646			
			L-S-Pe	[Pe-S]$^+$:565.5178; [L-Pe]$^+$:561.4742; [L-S]$^+$:603.5369			
			Po-S-H	[H-S]$^+$:591.5364; [Po-H]$^+$:561.4742; [Po-S]$^+$:577.5157			
12.363	860.7770	$C_{54}H_{102}NO_6^+$	O-L-Pe	[Pe-L]$^+$:561.4814; [O-L]$^+$:601.5345; [O-Pe]$^+$:563.5072	0.10 ± 0.00[a]	0.10 ± 0.02[a]	51:3
			O-H-Po	[Po-H]$^+$:561.4814; [O-Po]$^+$:575.5125; [O-H]$^+$:589.5167			
12.473	880.8372	$C_{55}H_{110}NO_6^+$	Ar-P-P	[P-P]$^+$:551.5081; [Ar-P]$^+$:607.5637	0.32 ± 0.02[a]	0.03 ± 0.00[b]	52:0
			S-S-P	[P-S]$^+$:579.5384; [S-S]$^+$:607.5637			
12.638	878.8212	$C_{55}H_{108}NO_6^+$	S-O-P	[P-O]$^+$:577.5182; [S-P]$^+$:579.5346; [S-O]$^+$:605.5522	0.28 ± 0.02[b]	0.90 ± 0.01[a]	52:1
			Ar-P-Po	[Po-P]$^+$:549.4811; [Ar-Po]$^+$:605.5522; [Ar-P]$^+$:607.5670			
			Go-P-P	[P-P]$^+$:551.5057; [Go-P]$^+$:605.5522			
12.710	876.8040	$C_{55}H_{106}NO_6^+$	O-P-O	[O-P]$^+$:577.5212; [O-O]$^+$:603.5396	4.09 ± 0.31[b]	6.44 ± 0.20[a]	52:2
			L-P-S	[S-P]$^+$:579.5291; [L-S]$^+$:603.5396; [L-P]$^+$:575.5015			
13.149	874.7872	$C_{55}H_{104}NO_6^+$	O-L-P	[P-L]$^+$:575.5043; [O-P]$^+$:577.5184; [O-L]$^+$:601.5167	0.55 ± 0.05[b]	1.03 ± 0.04[a]	52:3
			Di-P-P	[P-P]$^+$:551.4954; [Di-P]$^+$:601.5167			
			Ln-S-P	[P-S]$^+$:579.5158; [Ln-P]$^+$:573.4679; [Ln-S]$^+$:601.5167			
			S-L-Po	[Po-L]$^+$:573.4679; [S-Po]$^+$:577.5184; [S-L]$^+$:603.5374			
			O-O-Po	[Po-O]$^+$:575.5043; [O-O]$^+$:603.5374			
13.387	872.7766	$C_{55}H_{102}NO_6^+$	ARA-P-P	[P-P]$^+$:551.5228; [ARA-P]$^+$:599.5119	0.03 ± 0.00[b]	0.20 ± 0.01[a]	52:4
			O-L-Po	[Po-L]$^+$:573.4722; [O-Po]$^+$:575.4924; [O-L]$^+$:601.5225			
			O-Ln-P	[P-Ln]$^+$:573.4722; [O-P]$^+$:577.5264; [O-Ln]$^+$:599.5119			
			L-L-P	[P-L]$^+$:575.4924; [L-L]$^+$:599.5119			
13.476	890.8211	$C_{56}H_{108}NO_6^+$	No-Ma-H	[H-Ma]$^+$:577.5147; [No-H]$^+$:603.5295; [No-Ma]$^+$:605.5561	0.21 ± 0.01[b]	0.32 ± 0.00[a]	53:2
			O-O-Ma	[Ma-O]$^+$:591.5372; [O-O]$^+$:603.5295			
13.564	888.8082	$C_{56}H_{106}NO_6^+$	O-O-H	[H-O]$^+$:589.5126; [O-O]$^+$:603.5280	0.11 ± 0.01[a]	0.11 ± 0.01[a]	53:3
			O-L-Ma	[Ma-L]$^+$:589.5126; [O-Ma]$^+$:591.5402; [O-L]$^+$:601.5248			
13.624	906.8683	$C_{57}H_{112}NO_6^+$	Beh-P-Po	[Po-P]$^+$:549.4929; [Beh-Po]$^+$:633.5734; [Beh-P]$^+$:635.5895	0.19 ± 0.01[b]	0.28 ± 0.01[a]	54:1
			Er-P-P	[P-P]$^+$:551.4932; [Er-P]$^+$:633.5734			
			Ar-S-Po	[Po-S]$^+$:577.5233; [Ar-Po]$^+$:605.5491; [Ar-S]$^+$:635.5895			
			Ar-O-P	[P-O]$^+$:577.5233; [Ar-P]$^+$:607.5693; [Ar-O]$^+$:633.5734			
			Go-S-P	[P-S]$^+$:579.5262; [Go-P]$^+$:605.5491; [Go-S]$^+$:633.5734			
	904.8342		S-O-O	[O-O]$^+$:603.5375; [S-O]$^+$:605.5510	0.13 ± 0.01[b]		54:2

13.743		$C_{57}H_{110}NO_6^+$	L-S-S	$[S\text{-}S]^+$:607.5631; $[L\text{-}S]^+$:603.5375		0.39 ± 0.02[a]	
13.870	902.8211	$C_{57}H_{108}NO_6^+$	O-O-O	$[O\text{-}O]^+$:603.5378	0.39 ± 0.04[b]	2.61 ± 0.11[a]	54:3
14.309	902.8203	$C_{57}H_{108}NO_6^+$	S-O-L<br>Ln-S-S	$[L\text{-}O]^+$:601.5290; $[S\text{-}L]^+$:603.5383; $[S\text{-}O]^+$:605.5518<br>$[S\text{-}S]^+$:607.5652; $[Ln\text{-}S]^+$:601.5290	0.23 ± 0.01[b]	0.33 ± 0.00[a]	54:3
14.366	900.8062	$C_{57}H_{106}NO_6^+$	O-O-L<br>Ln-O-S<br>L-L-S	$[L\text{-}O]^+$:601.5163; $[O\text{-}O]^+$:603.5383<br>$[S\text{-}O]^+$:605.5476; $[Ln\text{-}S]^+$:601.5163; $[Ln\text{-}O]^+$:599.5190<br>$[S\text{-}L]^+$:603.5383; $[L\text{-}L]^+$:599.5190	0.18 ± 0.01[b]	2.02 ± 0.09[a]	54:4
14.856	898.7900	$C_{57}H_{104}NO_6^+$	Gln-O-O<br>ARA-O-P	$[O\text{-}O]^+$:603.5295; $[Gln\text{-}O]^+$:599.5087<br>$[P\text{-}O]^+$:577.5115; $[ARA\text{-}P]^+$:599.5087; $[ARA\text{-}O]^+$:625.5305	ND	0.04 ± 0.00	54:5

t_R, retention time (min); * GZG, Guanzhong goat milk; ** XSG, Xinong Saanen goat milk; ACN, acyl carbon number; DB, double bonds number; abbreviations of FAs used in structure of TAGs: Bu, butyric acid (C4:0); Co, caproic acid (C6:0); Cy, caprylic acid (C8:0); Ca, capric acid (C10:0); De, decenoic acid (C10:1); Ud, undecanoic acid (C11:0); La, lauric acid (C12:0); Lo, lauroleic acid (C12:1); Tr, tridecanoic acid (C13:0); M, myristic acid (C14:0); Mo, myristoleic acid (C14:1 n-5c); Pe, pentadecylic acid (C15:0); Pen, pentadecenoic acid (C15:1 n-5c); P, palmitic acid (C16:0); Po, palmitoleic acid (C16:1 n-7c); Ma, margaric acid (C17:0); H, heptadecenoic acid (C17:1 n-7t); S, stearic acid (C18:0); O, oleic acid (C18:1 n-9c); L, linoleic acid (C18:2 n-6c); Ln, alpha-linolenic acid (C18:3 n-3c); Gln, gamma-linolenic acid (C18:3 n-6c); N, nonadecanoic acid (C19:0), No, nonadecenoic acid (C19:1), Ar, arachidic acid (C20:0); Go, gadoleic acid (C20:1); ARA, arachidonic acid (C20:4 n-6c); Beh, behenic acid (C22:0); Er, erucic acid (C22:1). X–Y–Z: structure of TAGs, i.e., O-P-Cy stands for oleoyl-palmitoyl-capryloyl-glycerol structure; regiospecificity of the FAs in the TAGs was not determined. Results represent mean ± standard deviation, n =3. Presented values with the different superscript letters (within a row) are significantly different by independent samples *t*-test ($p \leq 0.05$). ND, not detected.
